# *Colocasia esculenta* Corm Extract Attenuates Ethanol-Induced Gastric Ulcer by Suppressing NF-κB Expression: An Integrated Network Pharmacology Approach and In Vivo Validation

**DOI:** 10.3390/ijms27177693

**Published:** 2026-08-27

**Authors:** Reza Pertiwi, Gofarana Wilar, Sri Adi Sumiwi, Jutti Levita

**Affiliations:** 1 Faculty of Pharmacy, Universitas Padjadjaran, Sumedang 45363, Indonesia; reza25001@mail.unpad.ac.id; 2 Faculty of Mathematics and Natural Sciences, Universitas Bengkulu, Bengkulu 38371, Indonesia; 3 Department of Pharmacology and Clinical Pharmacy, Faculty of Pharmacy, Universitas Padjadjaran, Sumedang 45363, Indonesia; g.wilar@unpad.ac.id (G.W.); sri.adi@unpad.ac.id (S.A.S.)

**Keywords:** *Colocasia esculenta* phenolics, gastroprotective activity, healthcare, natural resource, non-communicable disease, nuclear factor-kappaB p65, peptic ulcer disease

## Abstract

The corm of *Colocasia esculenta* (L.) Scott is widely used for food and treating gastric problems. However, its gastroprotective activity remains unexplored. This article aims to investigate the gastroprotective activity of the ethanol extract of *C. esculenta* corm (EECE) in ethanol-induced gastric ulcer in rats. EECE was analyzed for nutritional composition (proximate and vitamin C) and phytochemical composition (total phenol content, total flavonoid content, and quercetin levels), followed by metabolite profiling and an in vivo study. Male Wistar rats were randomly assigned to seven groups: (1) normal control, (2) negative control, (3) sucralfate group, (4) quercetin group, and three EECE groups at doses of (5) 200 mg/kg BW, (6) 400 mg/kg BW, and (7) 800 mg/kg BW. Following a two-week treatment period, all groups except the normal group were exposed to 70% ethanol. Post-mortem analysis included macroscopic and histopathological examination of gastric tissue, as well as Western blot analysis to investigate NF-κB p65 expression. EECE contains high moisture, ash, fat, and protein, and low carbohydrate levels, vitamin C at 417.73 mg/100 g, total phenol content of 833.43 mg GAE/100 g, total flavonoid content of 1178.82 mg QE/100 g, and quercetin at 261.76 mg/100 g. UHPLC–HRMS/MS analysis revealed amino sugars, conjugated amino acids, lipids, phenolics, and minor cyanogenic glycosides. EECE significantly reduced the percentage of ulcer area in an ethanol-induced gastric ulcer model (*p* < 0.05). It increased mucosal thickness, reduced polymorphonuclear cell infiltration, and decreased NF-κB p65 expression. The gastroprotective effects of EECE are mediated by enhancing gastric mucosal defense and inhibiting NF-κB p65 expression, thus highlighting its potential as a promising gastroprotective adjunct therapy.

## 1. Introduction

Peptic ulcer disease (PUD) remains a major global health problem with a steadily increasing prevalence. In 2019, PUD affected approximately 8.09 million individuals worldwide, representing a 25.82% increase since 1990 [1]. In Indonesia, the prevalence reaches 274,396 cases (40.8% of the population), with PUD-related deaths accounting for 0.13% of total deaths (https://www.worldlifeexpectancy.com/indonesia-peptic-ulcer-disease) (accessed on 14 December 2025). PUD is characterized by gastric mucosal damage caused by excessive gastric acid and pepsin secretion, which may progress to submucosal ulcer, gastric bleeding, and perforation [2]. Major risk factors associated with PUD include *Helicobacter pylori* infection, psychological stress, long-term use of non-steroidal anti-inflammatory drugs (NSAIDs), alcohol consumption, and oxidative stress, all of which play critical roles in disease pathogenesis [3,4].

Gastric mucosal protection and healing occur through two principal mechanisms: physical protection via the formation of a mucosal barrier and molecular mechanisms that suppress inflammatory responses, particularly those mediated by the NF-κB signalling pathway [5,6]. The synergistic interaction between these mechanisms is essential for maintaining mucosal integrity and accelerating the repair of damaged gastric tissue [7]. Accordingly, enhancing gastroprotective mechanisms by modulating inflammation-related signalling pathways has become a key therapeutic target in the management of PUD.

Current pharmacological therapies for PUD include antacids, histamine H_2_-receptor antagonists, mucosal protective agents, and proton pump inhibitors (PPIs) [8]. Although PPIs are generally effective, their long-term use has been associated with adverse effects such as renal dysfunction, cardiovascular disease, micronutrient deficiencies, and neurodegenerative disorders [9,10]. These concerns underscore the need for safer, more sustainable gastroprotective agents derived from natural sources [11].

Tuberous plants have been reported to exhibit gastroprotective properties due to their rich content of secondary metabolites, including flavonoids, tannins, alkaloids, and phenolic compounds, which possess antioxidant and anti-inflammatory activities [12,13,14]. Flavonoids contribute to gastric mucosal protection by stimulating mucus and bicarbonate secretion, exerting antioxidant effects, and inhibiting inflammatory responses and *H. pylori* infection [15,16]. In addition, tannins and alkaloids play important roles in mucosal defense by forming protective layers and reducing gastric acid secretion [17,18].

Taro (*Colocasia esculenta* L.) is a tuberous plant traditionally used for gastric disorders. It is known to contain a wide range of bioactive metabolites, including flavonoids, tannins, saponins, alkaloids, polysaccharides, and phenolic compounds [19,20,21,22]. Its corm has been reported to possess antioxidant and anti-inflammatory activities that protect the gastric mucosa against oxidative stress and inflammation, two key mechanisms underlying PUD pathogenesis [23,24,25]. Strong antioxidant capacity has also been demonstrated by DPPH and ABTS radical-scavenging assays, which are associated with its high phenolic and flavonoid content [26,27].

At the molecular level, flavonoids inhibit the PI3K/Akt/NF-κB signaling pathway, which plays a crucial role in the development of acid- or ethanol-induced gastric ulcers [6]. Quercetin, a major flavonoid, has been shown to reduce Akt phosphorylation and suppress NF-κB p65 expression, thereby exerting significant gastroprotective effects [28,29,30]. *C. esculenta* corm contains flavonoid compounds such as quercetin [31].

Despite these promising bioactivities, comprehensive studies evaluating the gastroprotective efficacy of *C. esculenta* corm extract in ethanol-induced gastric ulcer models—particularly those integrating metabolite profiling with the NF-κB p65 pathway—remain limited. Therefore, this study aimed to investigate the gastroprotective effects of the ethanol extract of *Colocasia esculenta* corm (EECE) in an ethanol-induced gastric ulcer model, with a specific focus on the NF-κB signaling pathway. The novelty of this study lies in its integrative mechanistic approach combining UHPLC–HRMS/MS-based metabolite profiling with in vivo histopathological and molecular evaluation of NF-κB p65 suppression, providing new insights into EECE as a multifunctional natural anti-ulcer agent.

## 2. Results

### 2.1. Proximate Composition and Vitamin C Content of EECE

Proximate analysis revealed clear compositional differences between *C. esculenta* corm powder and the ethanol extract (EECE) (Table 1). Compared with the raw powder, EECE had higher moisture, ash, lipid, and protein contents, while the carbohydrate content was lower. In addition, EECE contained a high level of vitamin C, at 417.73 mg/100 g extract.

### 2.2. Total Phenolic Content (TPC) and Total Flavonoid Content (TFC)

The TPC of the EECE was 833.43 mg gallic acid equivalent (GAE)/100 g extract, and the TFC was 1178.82 mg quercetin equivalent (QE)/100 g extract.

### 2.3. HPLC Analysis of Quercetin in EECE

**Figure 1:**
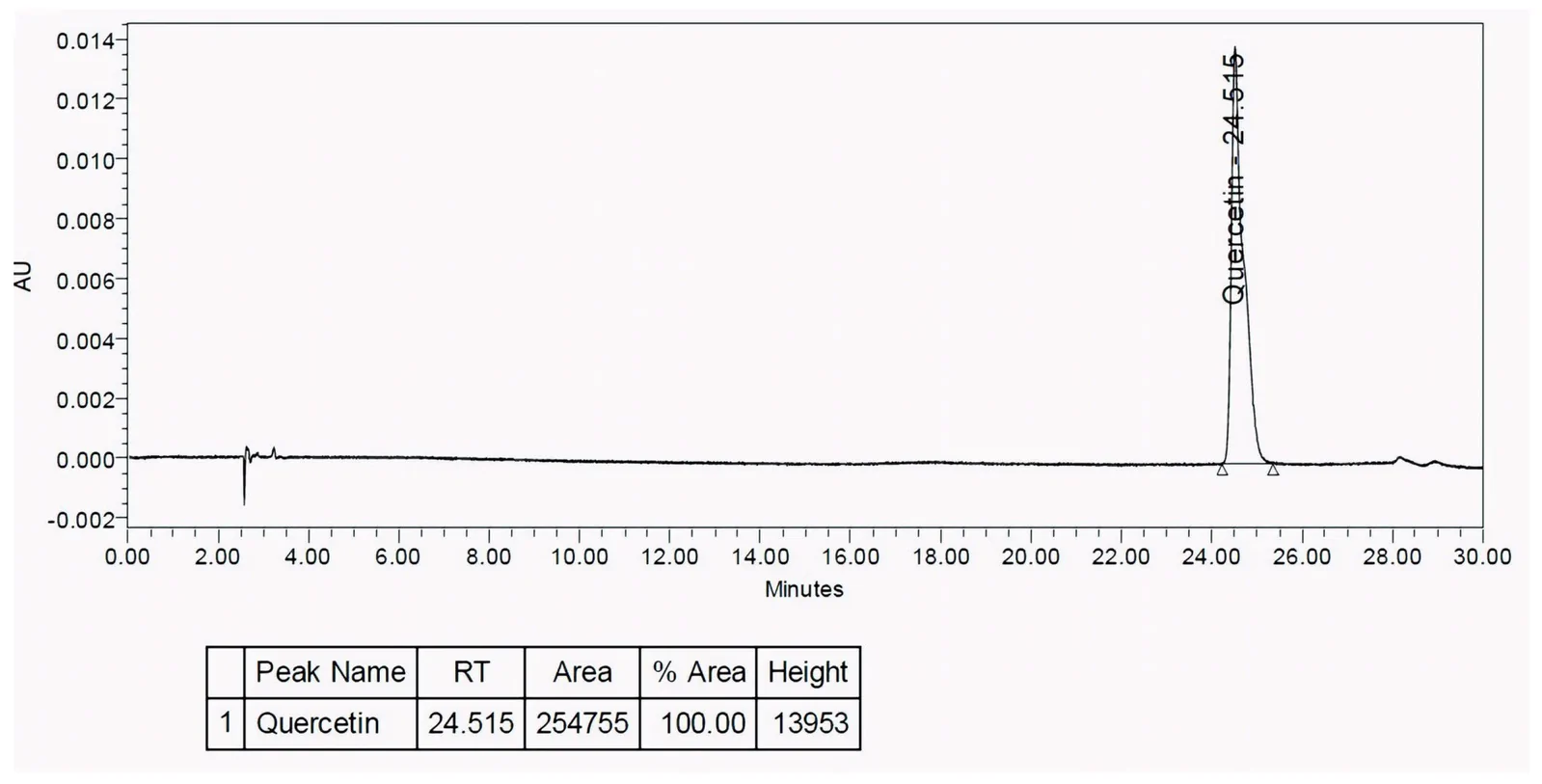
RP-HPLC chromatogram of quercetin standard, showing the quercetin peak at 24.515 min.

**Figure 2:**
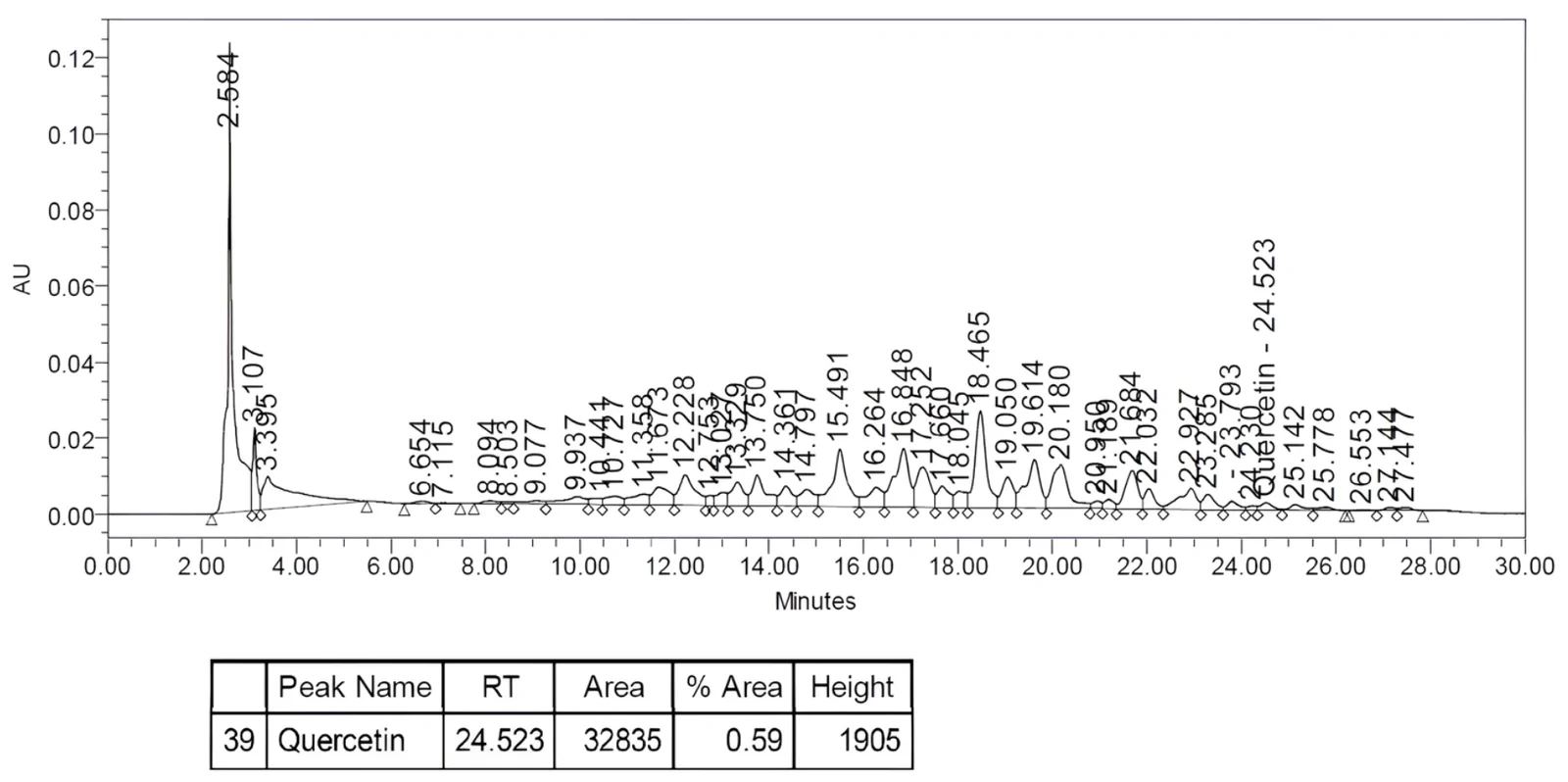
RP-HPLC chromatogram of EECE, showing the quercetin peak at 24.523 min.

Quercetin in EECE was identified and quantified by HPLC compared with a quercetin standard (2 ppm), which eluted at a retention time (RT) of 24.515 min (Figure 1). As expected, the RP-HPLC chromatogram of EECE showed numerous EECE metabolites, with quercetin observed at 24.523 min (Figure 2). Quantification was performed using a quercetin standard curve, yielding 261.76 mg per 100 g extract.

### 2.4. Metabolite Profiling and Putative Biological Implications of EECE

**Figure 3:**
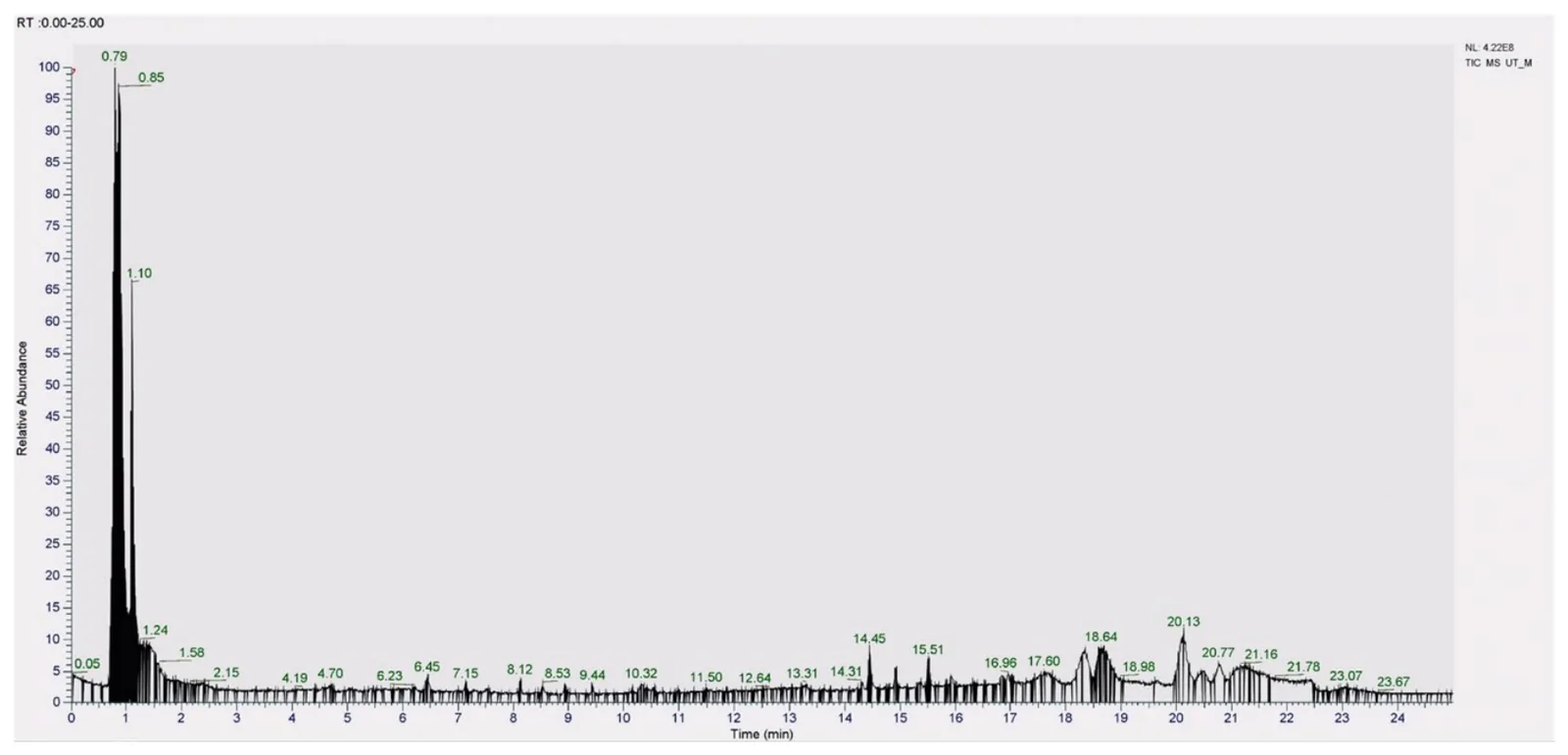
UHPLC chromatogram of EECE (T = 40 °C; flow rate = 0.3 mL min^−1^; sample: 1 mg mL^−1^ in MeOH 100%; injection volume: 3 µL).

To further investigate metabolites in EECE, a UHPLC–HRMS/MS system with electrospray ionization in positive mode (Figure 3) was employed, revealing major metabolite groups, which were summarized in Table 2. Metabolomic profiling revealed various putatively annotated metabolites belonging to several chemical classes, including amino acid derivatives, amino sugar derivatives, and lipid-related compounds. In UHPLC-HRMS/MS, the percentage relative abundance refers to the signal intensity of a specific ion, fragment, or metabolite scaled against the most intense peak (the base peak) in that specific mass spectrum or chromatogram, which is set to 100% [32]. The detailed information is provided in Supplementary Table S1.

### 2.5. Network Pharmacology-Based Mechanistic Insights

**Figure 4:**
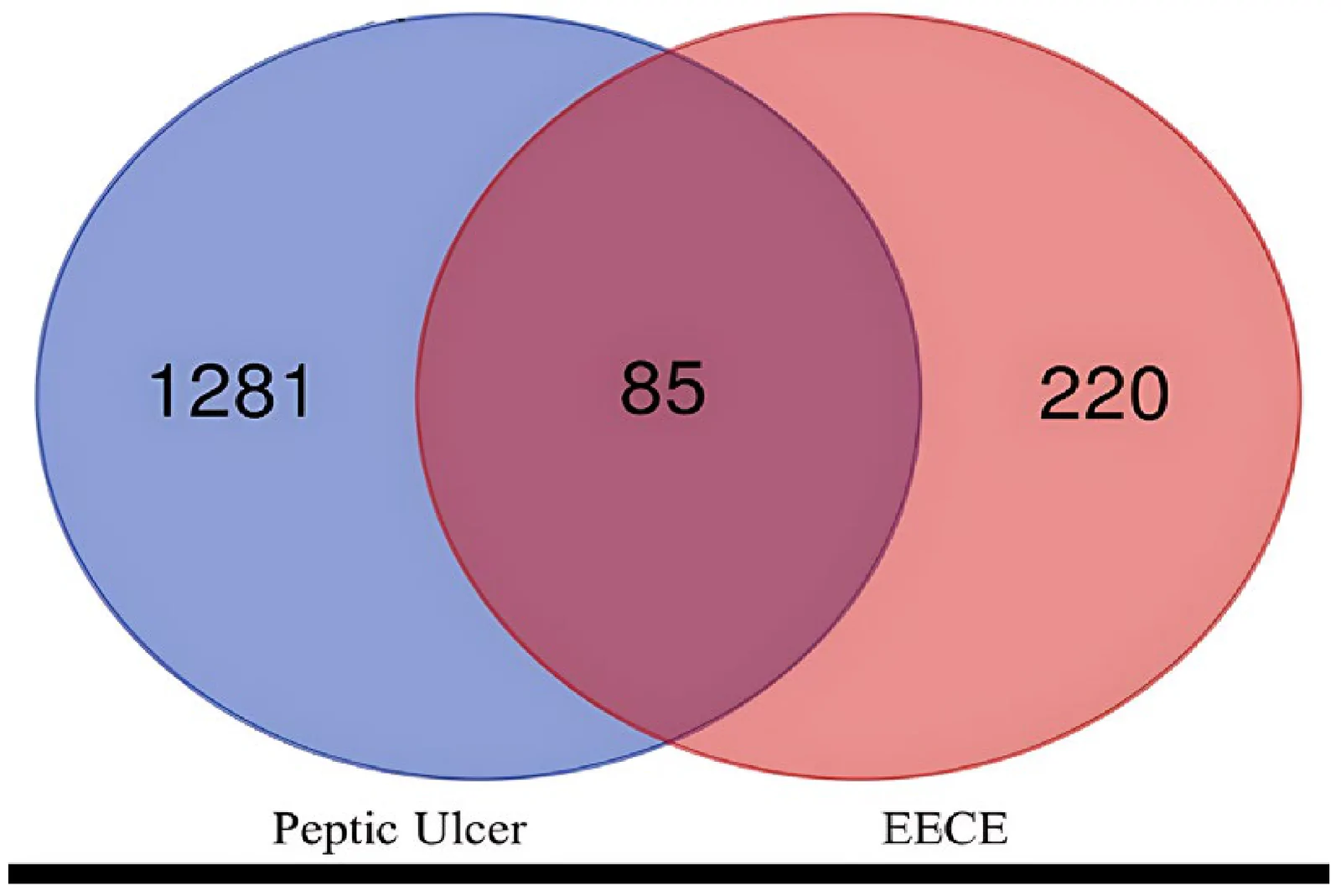
Venn diagram showing the intersection between EECE-related targets and PUD–associated targets retrieved from the GeneCards database. A total of 85 common targets were identified and subjected to subsequent network pharmacology analysis.

Putative gastroprotective activity of the most abundant EECE metabolites was predicted using network pharmacology to investigate the multi-compound, multi-target characteristics of EECE in the context of PUD. Compound annotation identified 34 putative compounds based on UPLC–HRMS analysis. Target prediction for compounds with available target information yielded 220 potential protein targets. In parallel, a total of 1281 PUD-related targets were retrieved from the GeneCards database. The predicted compound targets were then compared with the PUD-related targets to identify overlapping targets for subsequent network pharmacology analysis. Not all of the 34 annotated compounds showed associations with PUD-related targets; therefore, compounds without relevant overlapping targets were excluded from further analysis. An intersection analysis between compound-related and disease-associated targets identified 85 common targets (Figure 4).

**Figure 5:**
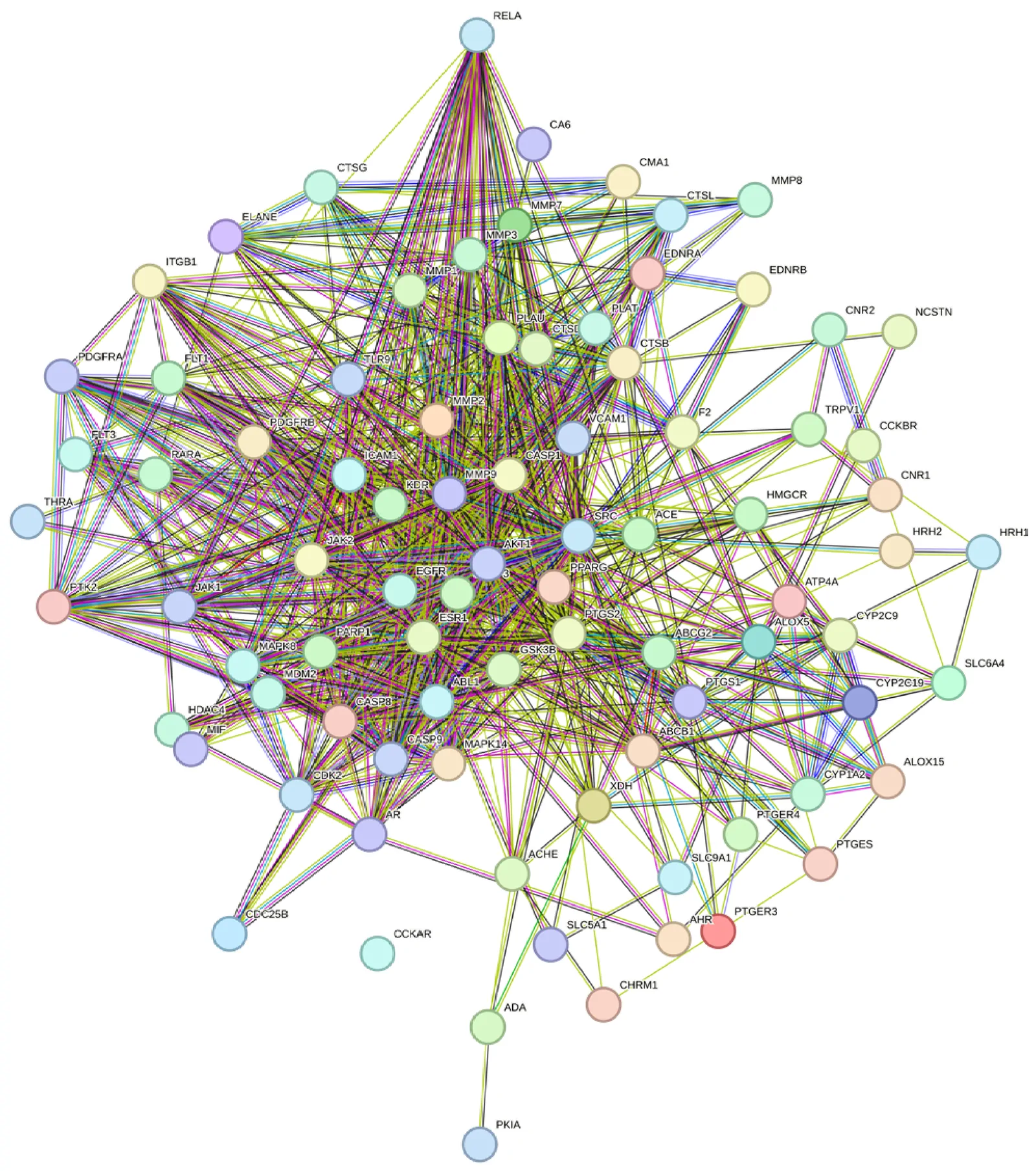
Protein–protein interaction (PPI) network of the intersecting targets constructed using the STRING database. Nodes represent proteins, and edges indicate protein–protein interactions.

**Figure 6:**
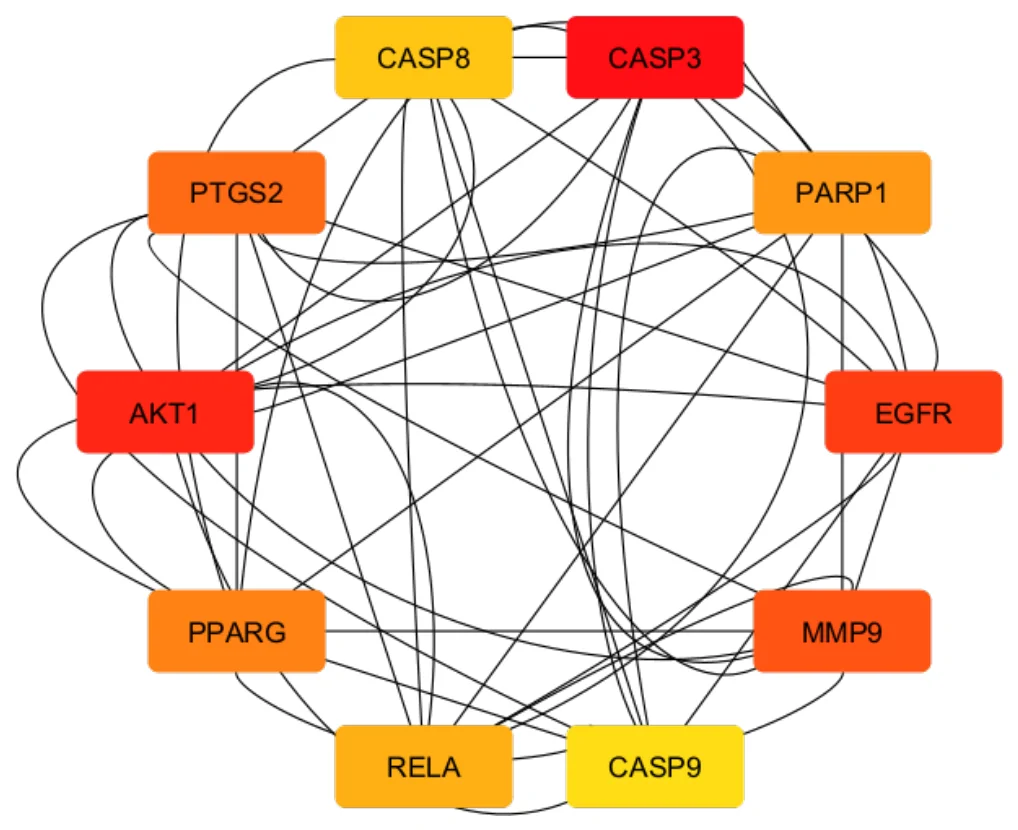
The top ten hub genes identified from the PPI network using the MCC method.

**Figure 7:**
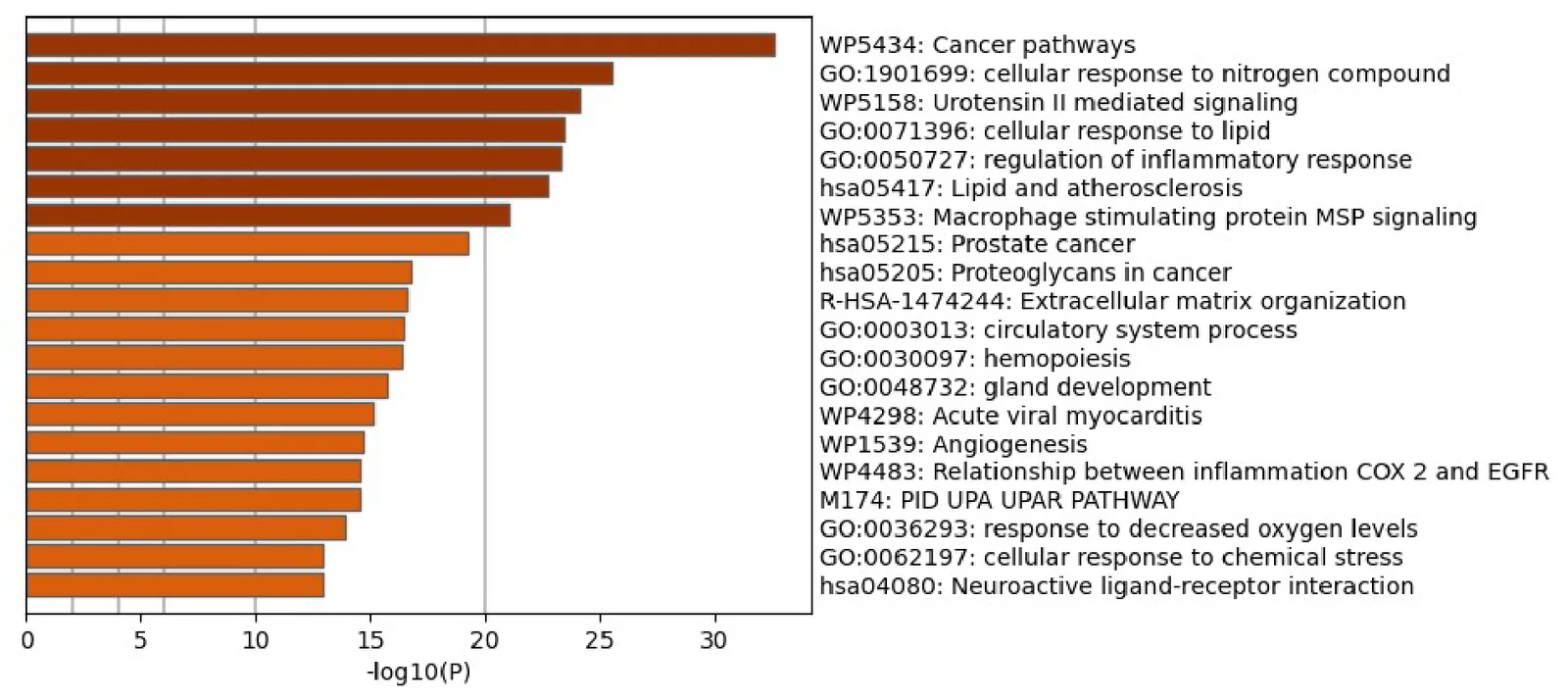
Bar chart of enriched Gene Ontology (GO) terms and biological pathways based on the intersecting targets. Bars are ranked by −log10(*p*), with higher values indicating greater statistical significance.

Protein–protein interaction (PPI) network analysis of the intersecting targets revealed a highly interconnected network (Figure 5). Using the Maximal Clique Centrality (MCC) algorithm implemented in CytoHubba, the top 10 hub genes were identified as CASP3, AKT1, EGFR, MMP9, PTGS2, PPARG, PARP1, RELA, CASP8, and CASP9 (Figure 6). GO enrichment and pathway analyses indicated that the intersecting targets were significantly enriched in multiple biological processes and signaling pathways related to peptic ulcer disease (Figure 7). The enrichment results demonstrated strong statistical significance, as indicated by −log10(*p*) values.

### 2.6. Macroscopic Evidence of Gastric Mucosal Protection by EECE

**Figure 8:**
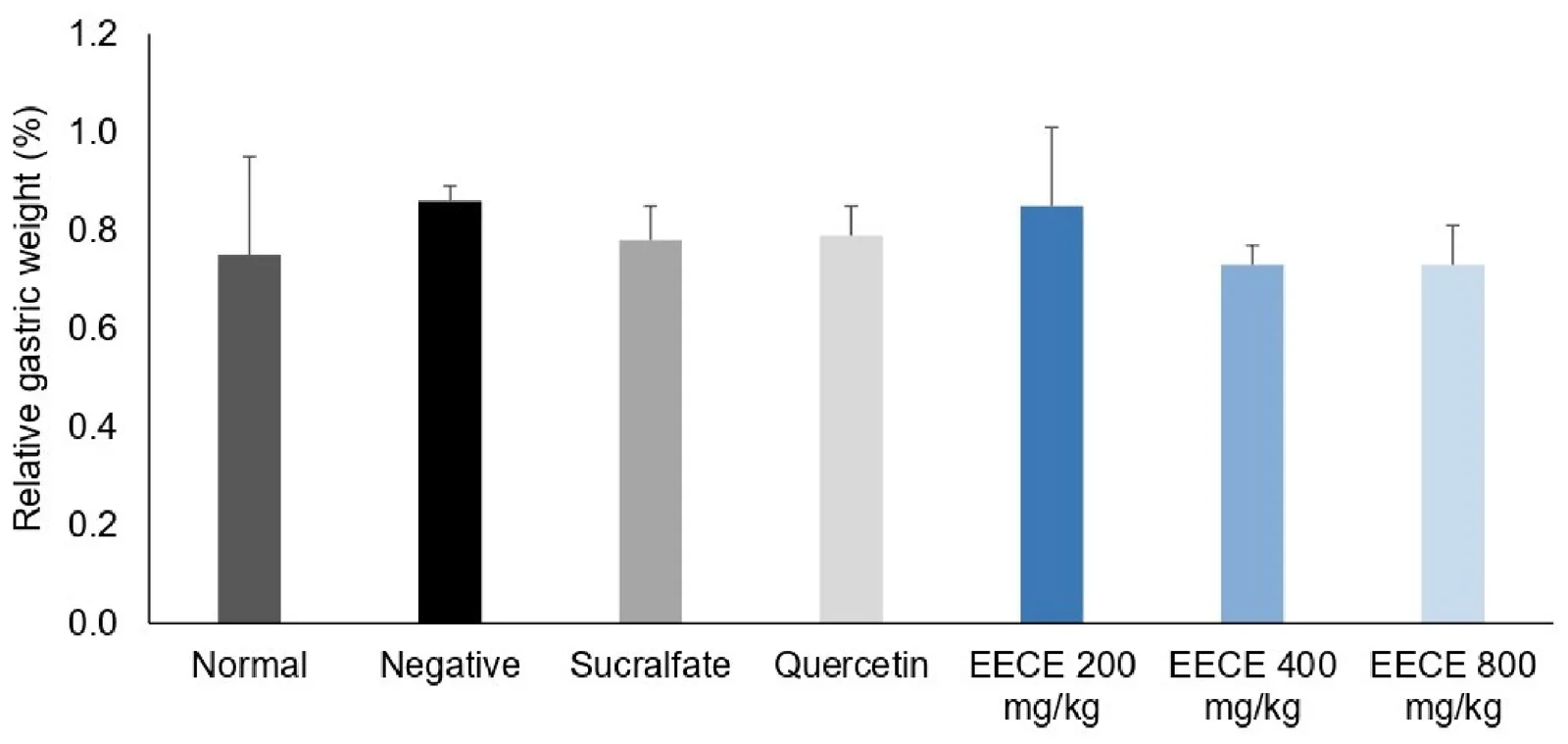
Relative gastric weight of rats with ethanol-induced gastric ulcer (*n* = 3). Values are expressed as mean ± SD (*n* = 3). Statistical analysis was performed using the Kruskal–Wallis test followed by the Mann–Whitney U test for post hoc pairwise comparisons.

Relative gastric weight was evaluated as an initial macroscopic parameter to assess gross gastric responses following ethanol exposure and EECE treatment [33,34]. As shown in Figure 8 and Table 3, no significant differences in relative gastric weight were observed among the experimental groups, indicating that EECE administration at all tested doses did not induce gross gastric enlargement or abnormal changes in tissue mass.

**Figure 9:**
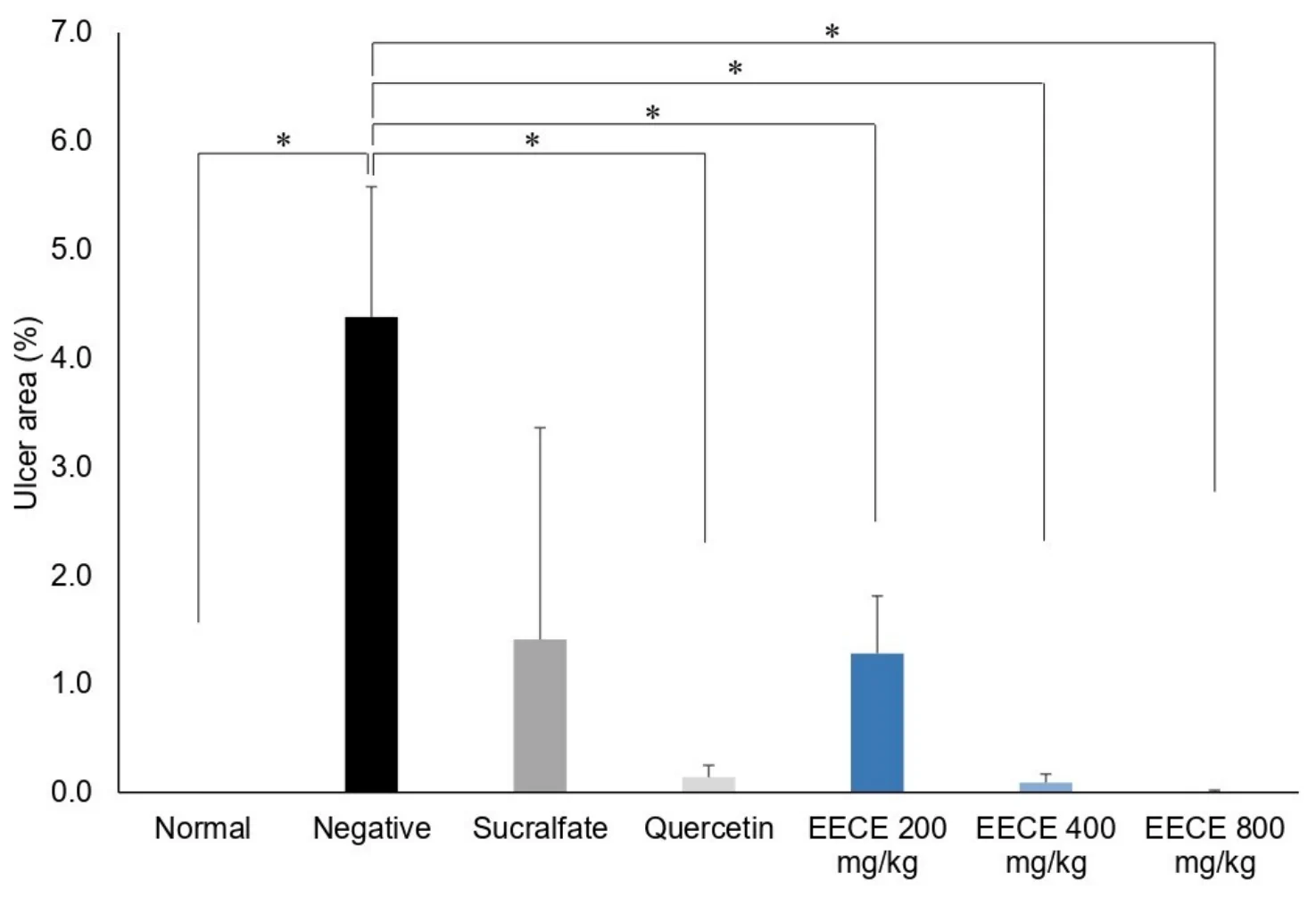
Ulcer area in rats with ethanol-induced gastric ulcer (*n* = 3). Values are expressed as mean ± SD (*n* = 3). Statistical analysis was performed using the Kruskal–Wallis test followed by the Mann–Whitney U test for post hoc pairwise comparisons. * indicates a statistically significant difference compared with the negative control group (*p* < 0.05).

**Figure 10:**
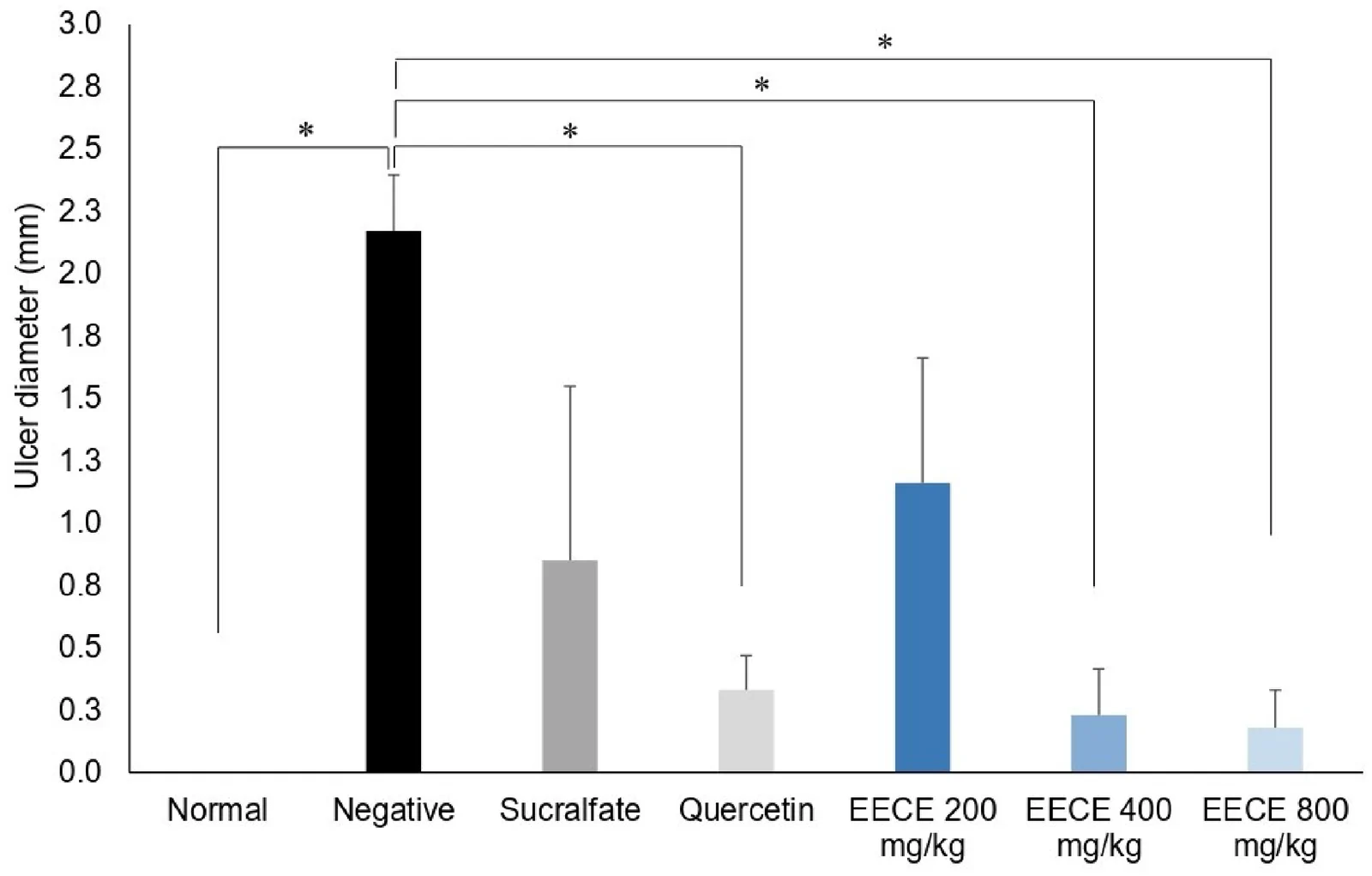
Ulcer diameter of rats with ethanol-induced gastric ulcer (*n* = 3). Values are expressed as mean ± SD (*n* = 3). Statistical analysis was performed using the Kruskal–Wallis test followed by the Mann–Whitney U test for post hoc pairwise comparisons. * indicates a statistically significant difference compared with the negative control group (*p* < 0.05).

**Figure 11:**
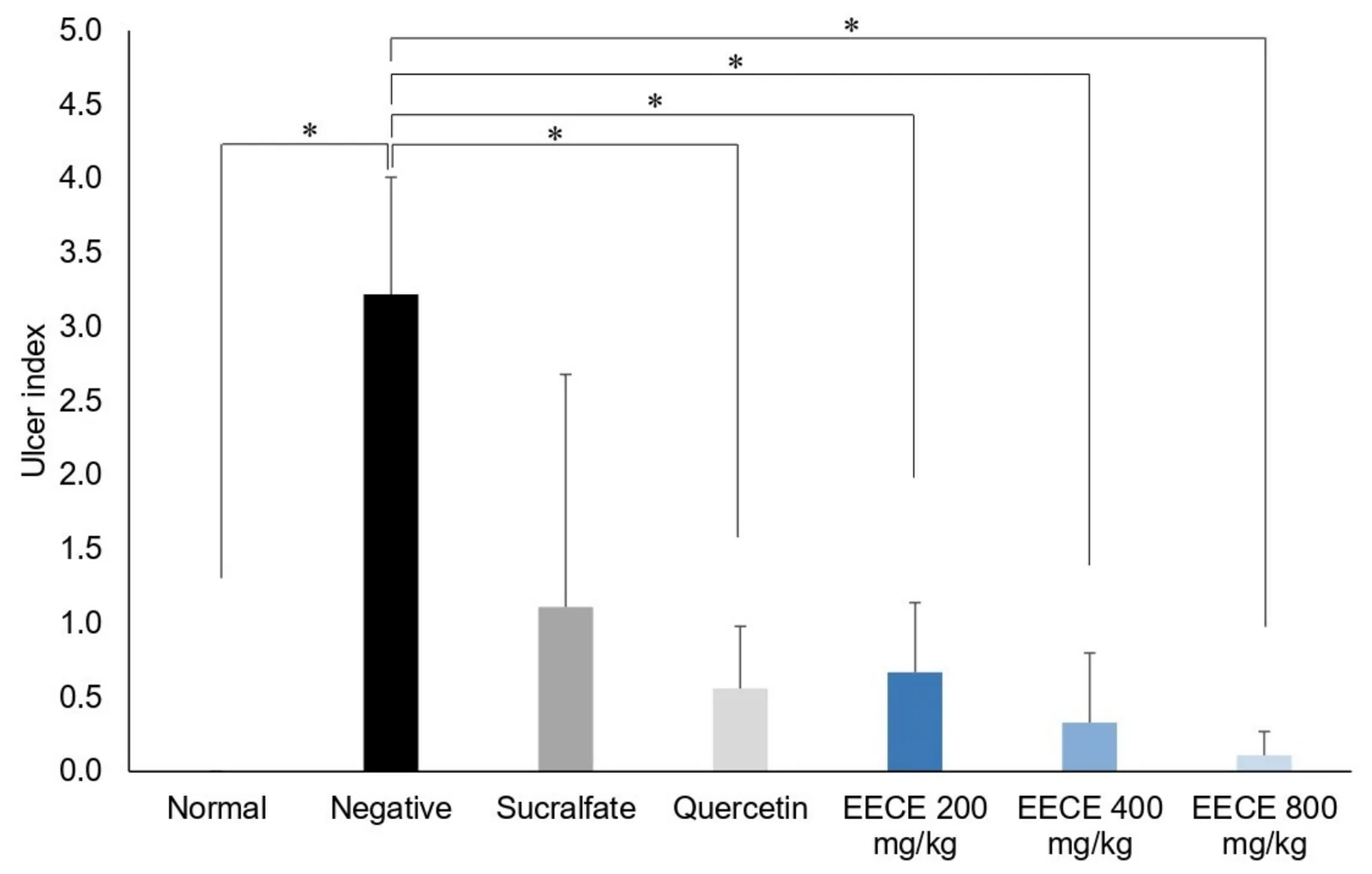
Ulcer index of rats with ethanol-induced gastric ulcer (*n* = 3). Values are expressed as mean ± SD (*n* = 3). Statistical analysis was performed using the Kruskal–Wallis test followed by the Mann–Whitney U test for post hoc pairwise comparisons. * indicates a statistically significant difference compared with the negative control group (*p* < 0.05).

**Figure 12:**
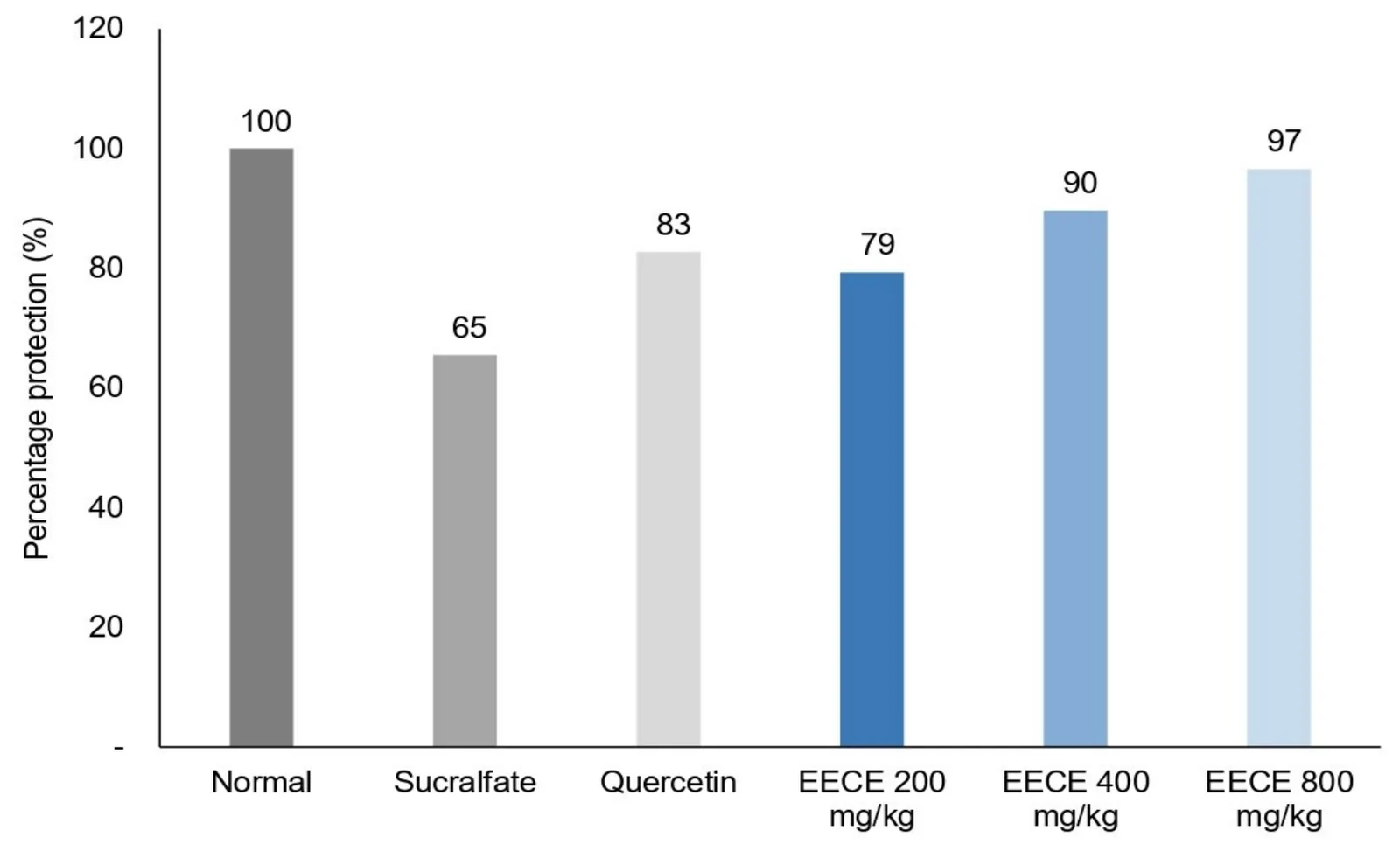
Percentage protection of rats with ethanol-induced gastric ulcer (*n* = 3).

**Figure 13:**
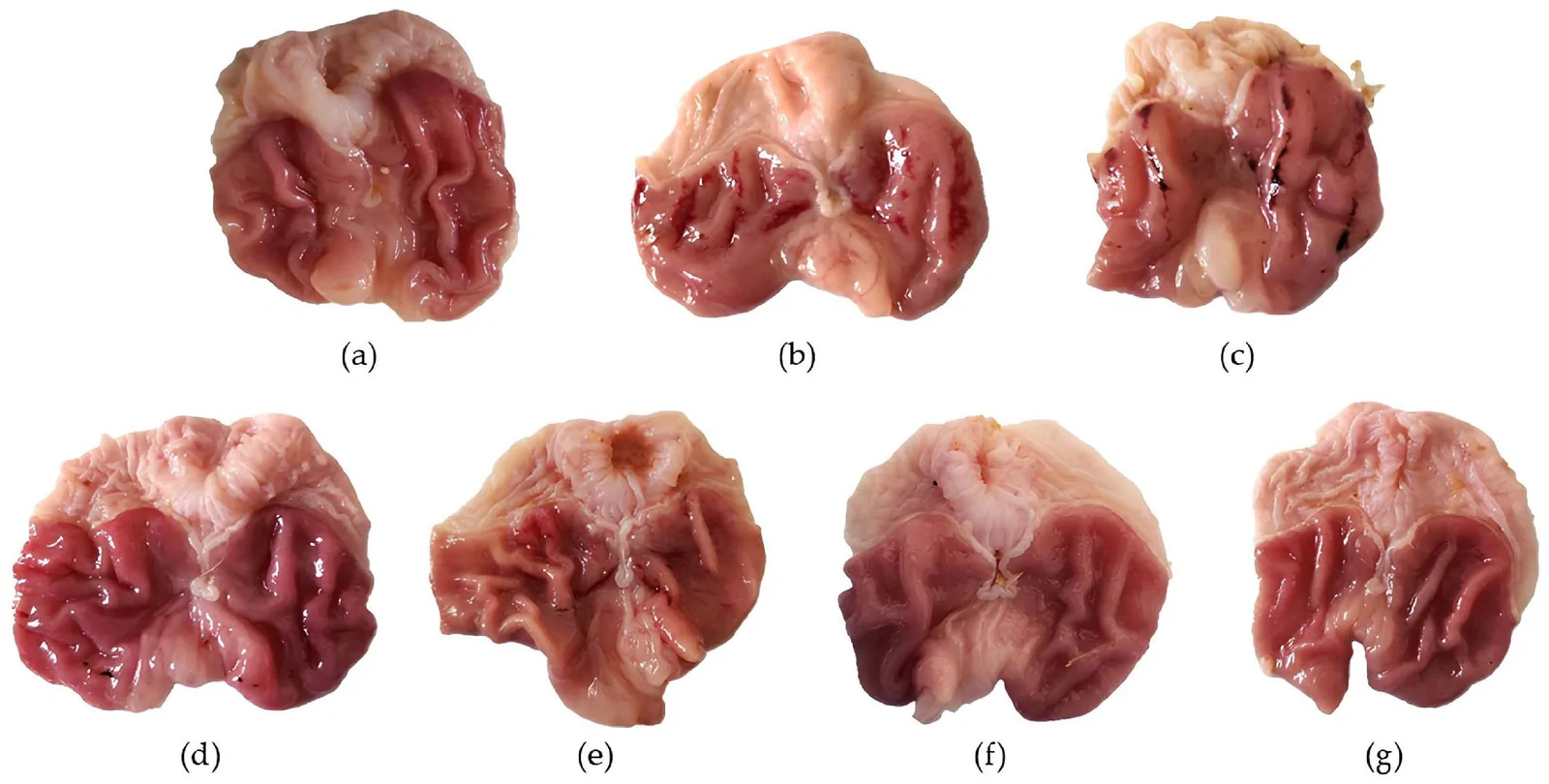
Macroscopic evaluation of gastric anti-ulcer effects. (**a**) Normal group; (**b**) negative control; (**c**) positive control treated with sucralfate; (**d**) positive control treated with quercetin; (**e**) EECE (200 mg/kg); (**f**) EECE (400 mg/kg); and (**g**) EECE (800 mg/kg).

In contrast, macroscopic indicators of gastric mucosal injury were markedly altered following ethanol administration (Table 3). The negative control group exhibited substantial increases in ulcer area (Figure 9), ulcer diameter (Figure 10), and ulcer index (Figure 11), accompanied by a marked reduction in percentage protection (Figure 12). The macroscopic evaluation of gastric tissues revealed clear differences in ulcer severity among the experimental groups, as shown in Figure 13. Conversely, treatment with EECE resulted in pronounced attenuation of these macroscopic lesions, as evidenced by reduced ulcer area, ulcer diameter, and ulcer index, together with an increased percentage of gastric protection, demonstrating clear macroscopic evidence of gastric mucosal protection by EECE.

### 2.7. Histopathological Scoring, Mucosal Thickness, and PMN Cell Infiltration in Gastric Tissue

Histopathological evaluation (Table 4) showed that the negative control group exhibited the most severe gastric damage, as indicated by the highest histopathological score (6.0 ± 1.6), a marked reduction in mucosal thickness (697.23 ± 91.41 µm), and a significant increase in polymorphonuclear neutrophil (PMN) infiltration (24.1 ± 0.5 cells/HPF). Morphologically, gastric tissues from this group demonstrated epithelial erosion, submucosal edema, and dense infiltration of acute inflammatory cells, predominantly neutrophils (PMNs).

In contrast, the normal group displayed minimal histopathological alterations, with a low damage score (1.0 ± 0.8), preserved mucosal thickness (1111.51 ± 136.73 µm), and a low PMN count (2.4 ± 1.2 cells/HPF). The sucralfate-treated group showed marked improvement compared with the negative control, as evidenced by a reduced histopathological score (1.9 ± 0.7), a significant decrease in PMN count (0.7 ± 0.1 cells/HPF; *p* < 0.05), and partial restoration of mucosal thickness (1002.04 ± 116.44 µm). Quercetin treatment resulted in a low histopathological score (1.3 ± 0.7), a significant reduction in PMN infiltration (7.1 ± 0.3 cells/HPF; *p* < 0.05), and mucosal thickness comparable to that of the normal group (1105.05 ± 41.84 µm).

**Figure 14:**
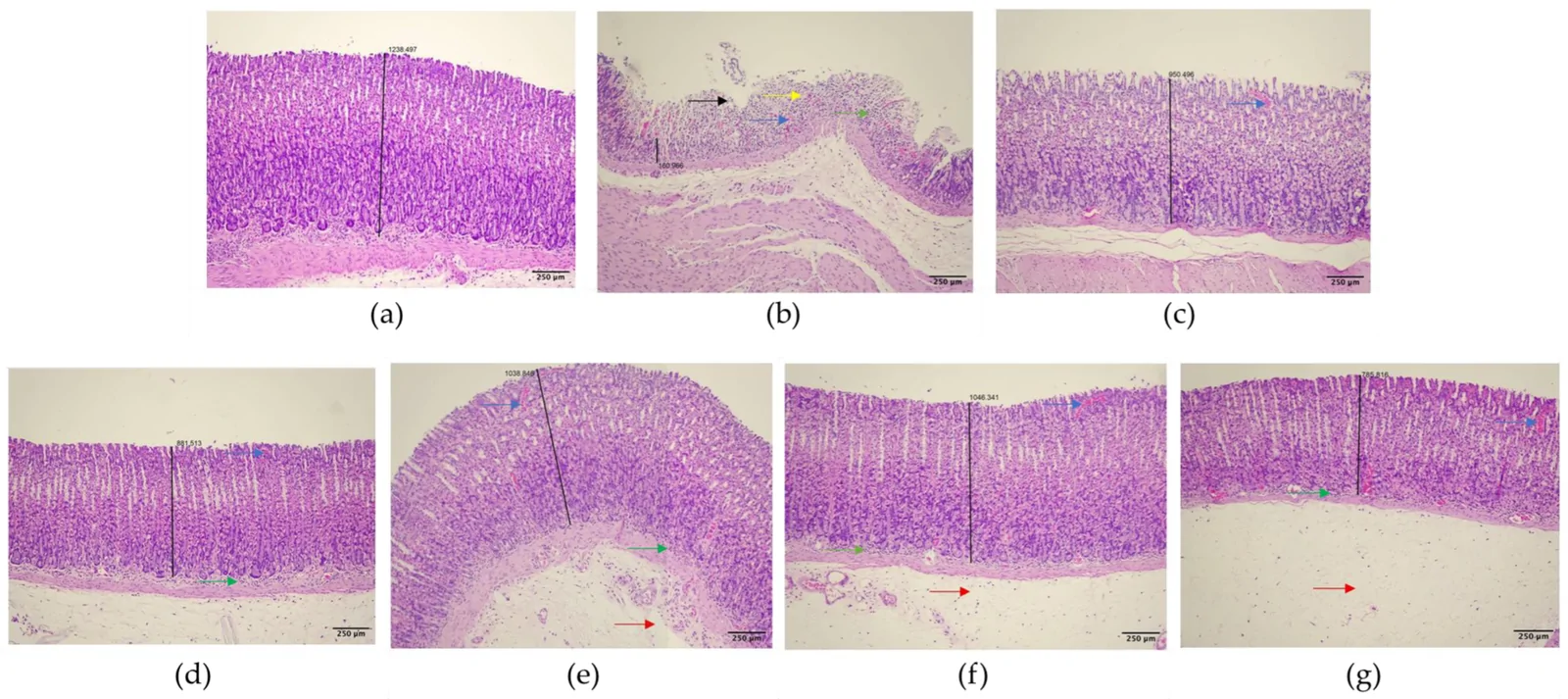
Histopathological examination of gastric tissue in experimental rats (100×). Representative hematoxylin and eosin (H&E)-stained sections of the gastric mucosa showing (**a**) normal control with intact epithelial lining, (**b**) ethanol-induced group, (**c**) sucralfate-treated group, (**d**) quercetin-treated group, and (**e**–**g**) EECE-treated groups. Black arrows indicate epithelial damage, red arrows indicate edema, blue arrows indicate hemorrhage, green arrows indicate inflammatory cell infiltration, and yellow arrows indicate epithelial erosion. Scale bar = 100 µm.

Administration of EECE at doses of 200, 400, and 800 mg/kg BW produced dose-dependent improvements in gastric histopathology. EECE at 200 mg/kg BW partially reduced the histopathological score (3.3 ± 1.3) and PMN count (10.6 ± 2.2 cells/HPF; *p* < 0.05), with mucosal thickness of 929.07 ± 38.32 µm. Increasing the dose to 400 mg/kg BW further improved histopathological parameters (score 2.5 ± 0.1; PMNs 10.2 ± 1.6 cells/HPF; *p* < 0.05), although mucosal thickness (1054.01 ± 35.69 µm) remained lower than that of the normal group. EECE at 800 mg/kg BW produced the most pronounced protective effect, as indicated by a low histopathological score (1.8 ± 1.0), a significant reduction in PMN infiltration (5.3 ± 0.9 cells/HPF; *p* < 0.05), and restoration of mucosal thickness (1061.71 ± 17.29 µm) approaching normal values. Representative histopathological images of gastric tissue are presented in Figure 14.

### 2.8. Effect of EECE on NF-κB p65 Expression

**Figure 15:**
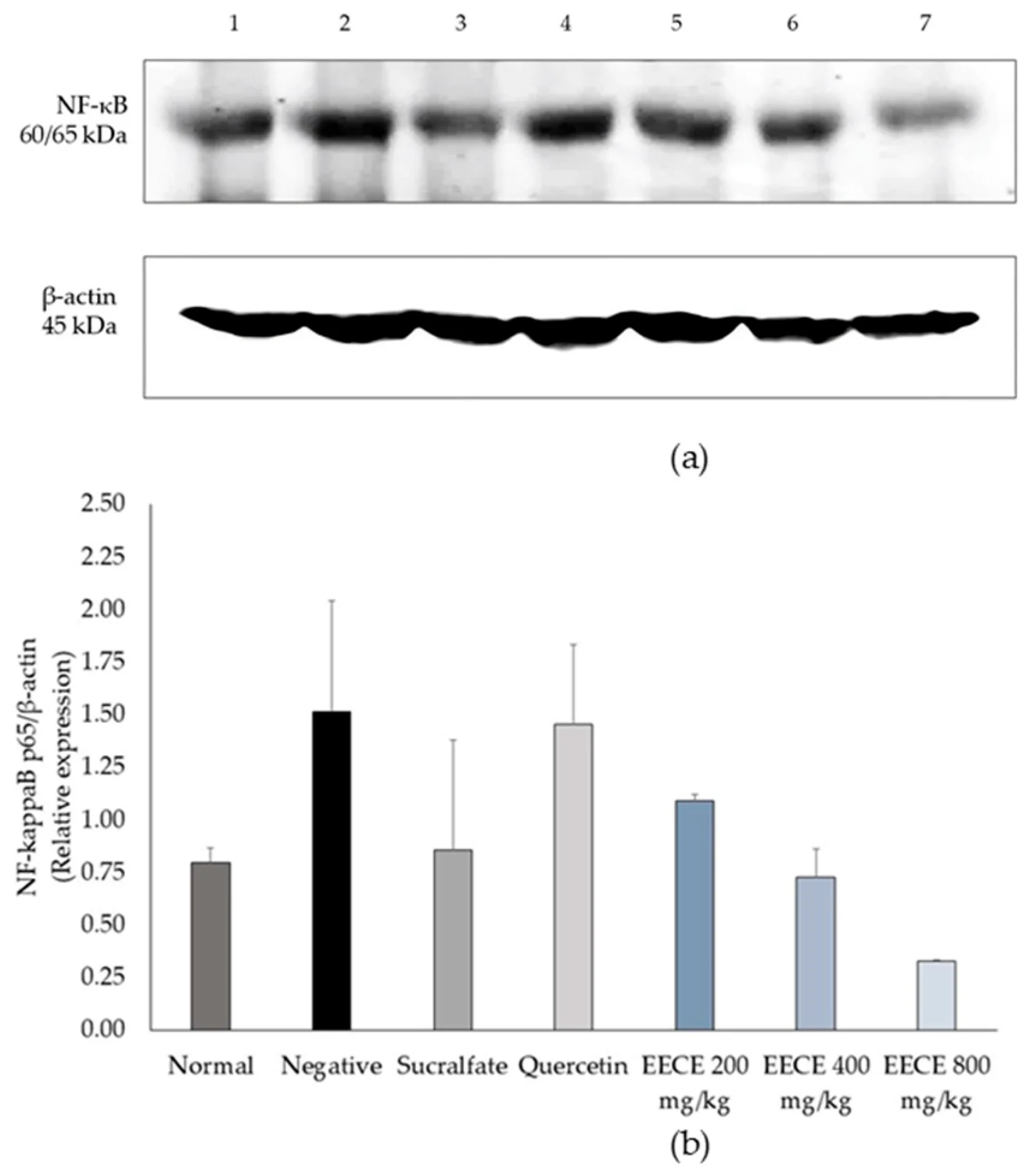
(**a**) Representative Western blot of NF-κB p65 expression. Representative Western blot bands showing the expression of NF-κB p65 protein in gastric tissues from the different experimental groups following ethanol-induced gastric injury; (**b**) Quantification of NF-κB p65 protein expression. β-Actin was used as the internal loading control. Note: 1, Normal; 2, Negative; 3, Sucralfate; 4, Quercetin; 5, EECE 200 mg/kg; 6, EECE 400 mg/kg; 7, EECE 800 mg/kg.

Western blot analysis (Figure 15) revealed differences in NF-κB p65 protein expression among experimental groups, which were further confirmed by quantitative analysis. The normal group exhibited low NF-κB p65 expression (0.79 ± 0.07). In contrast, ethanol induction in the negative control group resulted in increased NF-κB p65 expression (1.51 ± 0.53). In the treatment groups, NF-κB p65 expression was reduced relative to the negative control. The sucralfate and quercetin groups showed mean expression levels of 0.85 ± 0.52 and 1.46 ± 0.38, respectively.

Administration of EECE resulted in a dose-dependent reduction in NF-κB p65 expression, with mean values of 1.09 ± 0.03 at 200 mg/kg BW, 0.73 ± 0.13 at 400 mg/kg BW, and 0.33 ± 0.00 at 800 mg/kg BW. Although differences in NF-κB p65 expression among groups did not reach statistical significance (*p* > 0.05), a consistent downward trend was observed as EECE dose increased.

## 3. Discussion

The present study demonstrates that the gastroprotective effects of EECE are closely associated with its chemical composition, which collectively modulates oxidative stress, inflammatory signaling, and mucosal integrity in the ethanol-induced gastric ulcer model. Ethanol-induced gastric injury is a multifactorial process involving direct epithelial disruption, excessive reactive oxygen species (ROS) generation, activation of inflammatory cascades, and impairment of mucosal defense mechanisms [35,36]. Therefore, effective gastroprotection requires simultaneous modulation of multiple biological targets rather than reliance on a single mechanism.

The hydroethanolic extraction procedure employed in the present study was selected to recover a broad spectrum of phytochemical constituents suitable for subsequent chemical characterization, network pharmacology analysis, and biological evaluation. Furthermore, sun-drying was employed as a practical post-harvest pretreatment; however, uncontrolled environmental conditions, including temperature, humidity, and sunlight exposure, may influence the stability of heat- and light-sensitive phytochemicals. Therefore, degradation of some labile constituents cannot be completely excluded. Importantly, all chemical analyses and biological experiments were performed using the same extract batch prepared under identical laboratory conditions, thereby minimizing experimental variability throughout the study. Future studies should incorporate controlled drying conditions, optimization of extraction parameters, and independent batch validation to further strengthen the robustness and reproducibility of the phytochemical characterization and biological findings.

Proximate analysis revealed a substantial compositional shift between *C. esculenta* corm powder and EECE, indicating that 70% ethanol selectively enriches polar and semi-polar constituents while excluding insoluble macromolecules, particularly starch, which dominates the native corm matrix. The drastic decrease in carbohydrates confirms that ethanol-insoluble polysaccharides largely remained in the residual solid phase, whereas extractable organic compounds and mineral-associated fractions became proportionally concentrated in the dried extract [37]. This compositional shift is biologically relevant, as carbohydrate depletion does not compromise gastroprotection, while enrichment of antioxidant and anti-inflammatory constituents directly targets the key pathological drivers of ethanol-induced mucosal injury. The total ash content of EECE was substantially higher than that of the raw corm powder. Total ash represents the inorganic residue, specifically the metal oxides, remaining after complete incineration of the sample. This increase may reflect the relative enrichment of extractable inorganic constituents in the dried extract after removal of a substantial portion of the original organic matrix during hydroethanolic extraction and concentration. However, because elemental analysis was not performed, the specific inorganic constituents contributing to the high ash value could not be determined.

In parallel, EECE exhibited a high vitamin C content (417.73 mg/100 g extract), highlighting the effectiveness of hydroethanolic extraction in preserving hydrophilic antioxidants. Vitamin C is a potent ROS scavenger and plays a crucial role in limiting oxidative damage to gastric epithelial cells. Excessive ROS generation following ethanol exposure is known to trigger lipid peroxidation, mitochondrial dysfunction, and redox-sensitive activation of NF-κB signaling [38,39]. Thus, the high vitamin C content of EECE likely contributes to early suppression of oxidative stress, thereby attenuating upstream activation of inflammatory cascades.

The antioxidant capacity of EECE is further supported by its elevated TPC and TFC. Phenolic compounds and flavonoids are well documented to exert gastroprotective effects through ROS scavenging, inhibition of lipid peroxidation, and modulation of inflammatory signaling pathways, including NF-κB [40,41]. In comparison, Akyüz (2019) [26] reported a lower TPC value of 240 mg GAE/100 g extract for an ethanolic extract of *C. esculenta* corms (Gölevez variety), highlighting the superior phenolic recovery achieved in the present study. Conversely, Nugroho et al. (2025) reported a markedly higher TPC value (3975 mg/100 g) for *C. esculenta* extracted using water [42], suggesting that extraction efficiency may be substantially enhanced through optimization of extraction parameters, such as solvent polarity, temperature, extraction duration, or the application of intensification techniques (e.g., sonication). Variations in TFC among studies are commonly attributed to differences in plant genotype, environmental growth conditions, post-harvest handling, and extraction and analytical methodologies [43,44,45,46]. Although previous studies have reported higher total phenolic content using aqueous extraction, hydroethanolic extraction was selected because its intermediate polarity enables the extraction of a broader range of phytochemical classes, including flavonoids, phenolic compounds, alkaloids, fatty acid derivatives, and other moderately polar metabolites required for comprehensive metabolomic profiling and subsequent network pharmacology analysis.

Untargeted UHPLC–HRMS/MS profiling revealed a chemically diverse metabolite composition in EECE, including lipid-derived metabolites, aromatic compounds, phenolic derivatives, and indole-related molecules. Lipid-associated metabolites such as oleamide and related fatty acid amides have been reported to exert anti-inflammatory effects by suppressing iNOS and COX-2 expression and modulating NF-κB signaling [47,48]. In addition, lipid components contribute to maintaining gastric mucus hydrophobicity, which is essential for preventing hydrogen-ion back-diffusion and epithelial injury [49].

Metabolite annotation was performed by integrating chromatographic retention time, accurate precursor mass, experimental MS/MS fragmentation spectra, and database-assisted spectral matching using the mzCloud Mass Spectral Library, with candidate structures subsequently cross-checked using the ChemSpider and PubChem databases. Nevertheless, the reported metabolite identities should be regarded as putative annotations, as authentic reference standards, retention-time confirmation with reference compounds, and quantitative spectral matching scores were not available. Consequently, although the combined use of accurate mass, MS/MS fragmentation patterns, and database-assisted spectral matching improves confidence in metabolite annotation, unequivocal structural confirmation cannot be claimed. Therefore, the subsequent network pharmacology analysis should be interpreted as a hypothesis-generating approach, and the predicted compound–target interactions require further experimental validation.

The presence of phenolic and indole derivatives, including trans-3-indoleacrylic acid, further supports the antioxidant potential of EECE. Even at relatively low concentrations, such compounds can synergistically attenuate oxidative stress and inflammatory amplification in ethanol-induced gastric injury [50]. Moreover, UHPLC–HRMS/MS analysis yielded putative annotations of several amino sugar-related metabolites, including N-Acetylquinovosamine and N-acetylglucosaminitol, which are structurally related to N-acetylglucosamine, a key precursor for mucin biosynthesis. Gastric mucus is a primary defensive barrier against ethanol-induced damage, and enhanced mucin production has been shown to improve mucosal resistance and ulcer healing [51,52].

The presence of aromatic metabolites such as 3-Indoleacrylic acid and other phenolic derivatives indicates active phenolic biosynthetic pathways that may contribute to the extract’s antioxidant potential [40]. The detection of compounds such as lotaustralin further suggests the presence of low-level cyanogenic glycosides, which are widely reported as characteristic defense-related metabolites in Araceae [53,54]. Overall, this complex metabolite profile supports the interpretation that EECE contains not only phenolic compounds quantified by total phenolic and flavonoid content analyses, but also other metabolite classes that may collectively contribute to its bioactive properties, including antioxidant, anti-inflammatory, and metabolic-modulatory activities [55,56].

Determining TFC and TPC in EECE provides initial quantitative insight into its chemical composition. EECE exhibited a considerable TFC, indicating that flavonoids constitute an important class of secondary metabolites in the extract. This finding is consistent with UHPLC–HRMS/MS analysis, which revealed multiple aromatic metabolites, phenolic derivatives, and indole compounds closely linked to flavonoid biosynthetic pathways in plants. Furthermore, the TPC value reflects the contribution of phenolic acids, aromatic indoles, and benzenoid derivatives detected through UHPLC–HRMS/MS. These results confirm that EECE contains not only flavonoids but also a broad spectrum of non-flavonoid phenolic compounds, in agreement with the characteristic chemical profile of corm-derived plant materials.

Supporting the contribution of phenolic constituents, HPLC analysis identified and quantified quercetin, a representative flavonol present in EECE. Quercetin is biochemically recognized for its strong capacity to scavenge reactive oxygen species and free radicals through both direct neutralization and modulation of endogenous antioxidant systems, thereby contributing to redox stabilization and attenuation of oxidative stress [57,58]. In addition to quercetin, UHPLC–HRMS/MS analysis revealed several putative annotated phenolic metabolites that may collectively contribute to the overall antioxidant potential of the extract. However, the individual contributions and possible interactions among these metabolites were not investigated in the present study; therefore, any synergistic effects should be regarded as hypothetical and require further experimental validation.

Although HPLC confirmed the presence of quercetin in EECE, this compound was not explicitly annotated in the untargeted UHPLC–HRMS/MS dataset. This discrepancy can be attributed to fundamental differences between targeted and untargeted analytical approaches. HPLC is a targeted technique used to selectively detect and quantify predefined analytes via retention-time matching and authentic reference standards [2,59]. In contrast, UHPLC–HRMS/MS employs an untargeted strategy that broadly detects metabolites without prior target selection, followed by annotation based on accurate mass, ionization behaviour, and spectral database matching [2,60]. Consequently, compounds confirmed by targeted HPLC analysis are not necessarily annotated during untargeted metabolomic profiling.

In addition to methodological factors, the ionization behavior of phenolic compounds is critical to their detectability by LC–MS-based techniques. Flavonols, such as quercetin, ionize more efficiently in negative electrospray ionization (ESI–) mode, forming stable deprotonated [M–H]^−^ ions that enhance detection sensitivity in high-resolution mass spectrometry. Previous studies employing LC–MS analyses in ESI negative-ion mode have demonstrated improved detection of flavonoid aglycones and related phenolic compounds under such conditions, supporting the preferential detection of quercetin-type metabolites in ESI–mode [31].

Moreover, the chemical form of quercetin in plant tissues further influences its detectability. In plants, quercetin predominantly occurs as flavonoid glycosides (e.g., quercetin-3-O-glucoside, quercetin-3-O-rutinoside, and quercetin-3-O-rhamnoside) rather than as a free aglycone. These glycosylated forms represent the dominant natural state of quercetin and significantly affect its ionization efficiency and bioavailability [61]. Consequently, the aglycone form may exhibit low signal intensity or remain undetected in untargeted UHPLC–HRMS/MS profiling, which preferentially captures more stable and readily ionizable glycosides [62]. Under the analytical conditions used in the present study, quercetin aglycone and its glycosides may have exhibited relatively low ionization efficiency, insufficient signal intensity, or limited database match, resulting in the absence of explicit annotation in the untargeted UHPLC–HRMS/MS dataset. This interpretation is supported by the relatively low quercetin concentration quantified by HPLC (261.76 mg/100 g extract), suggesting that quercetin may be present at levels close to the analytical detection or annotation threshold of the untargeted metabolomic workflow.

More broadly, this finding reflects an inherent limitation of the untargeted metabolomics approach used in this study. Metabolite identification was based on database matching of *m*/*z* and MS/MS fragmentation data without the use of authentic reference standards. Therefore, the reported metabolites should be regarded as putative annotations rather than unequivocally identified compounds. Consequently, the subsequent network pharmacology analysis should be interpreted as a hypothesis-generating approach, and the predicted compound–target interactions require further experimental validation.

Although untargeted metabolomic profiling may not comprehensively represent the complete phytochemical composition of EECE, the annotated metabolites obtained under the present analytical conditions provide a biologically relevant dataset for subsequent computational analyses. The annotated metabolite profile identified multiple putative bioactive compounds that may collectively contribute to the gastroprotective activity of EECE, suggesting potentially complex molecular interactions. To generate hypotheses regarding the molecular mechanisms underlying these biological effects, a network pharmacology analysis was performed to predict compound–target–pathway relationships. This analysis identified several hub genes, including RELA (NF-κB p65), AKT1, EGFR, PTGS2, MMP9, CASP3, CASP8, CASP9, and PARP1, as central regulators within the protein–protein interaction network. The prominence of these hub genes suggests that coordinated regulation of inflammatory signaling, cell survival, apoptosis, and tissue remodeling may contribute to the gastroprotective effects of EECE. However, these interactions represent computational predictions derived from network pharmacology and require experimental validation.

Among these hub genes, RELA (NF-κB p65) emerged as a prominent predicted regulatory node, suggesting a potential contribution of NF-κB-mediated signaling in gastric mucosal injury and repair. NF-κB p65 functions as a master transcription factor that regulates the expression of pro-inflammatory cytokines, including TNF-α, IL-1β, and IL-6, which are known to exacerbate gastric inflammation and epithelial damage [63,64]. Dysregulation of NF-κB signaling has been widely implicated in gastric mucosal injury, in which exposure to proinflammatory agents, such as ethanol, induces NF-κB p65 activation and nuclear translocation, amplifying inflammatory cascades and promoting epithelial damage [65].

GO enrichment analysis further demonstrated significant enrichment of biological processes related to the regulation of inflammatory response, cellular response to chemical stress, and response to nitrogen-containing compounds. These enriched biological processes are consistent with pathways regulated by NF-κB signaling, supporting the potential involvement of NF-κB p65 as an upstream regulator linking chemical stress exposure to inflammatory damage in gastric tissues. Moreover, enrichment of biological processes associated with extracellular matrix organization, angiogenesis, and gland development indicates the involvement of tissue remodeling and mucosal regeneration during peptic ulcer healing. These regenerative events may be associated with NF-κB signaling through downstream effectors such as matrix metalloproteinase-9 (MMP-9), which plays a key role in extracellular matrix degradation and remodeling. Increasing evidence suggests that excessive activation of the NF-κB–MMP-9 axis contributes to impaired ulcer healing, sustained inflammation, and persistent mucosal injury, whereas controlled modulation of this pathway promotes angiogenesis, epithelial restitution, and restoration of gastric mucosal integrity [66,67,68,69].

Pathway enrichment analysis indicated significant involvement of COX-2–EGFR signaling, receptor-mediated pathways, and lipid mediator–related signaling cascades. RELA (NF-κB p65) functions as a key upstream transcriptional regulator of COX-2 expression, thereby enhancing prostaglandin-mediated lipid signaling and facilitating EGFR transactivation [63]. Prostaglandin E_2_ signaling has been shown to play an essential role in epithelial cell survival, proliferation, and restitution processes [70]. Collectively, this coordinated NF-κB–COX-2/PGE_2_–EGFR crosstalk has been increasingly recognized as a central regulatory mechanism governing the balance between gastric mucosal injury and repair [71]. Consistent with previous studies, the identification of AKT1, EGFR, RELA, and MMP9 as predicted hub genes suggests that an NF-κB–AKT–EGFR signaling axis may contribute to the molecular mechanisms of gastric ulcer pathogenesis. Nevertheless, this proposed signaling network represents a computational prediction and requires experimental validation.

Although several enriched pathways were annotated as cancer-related, these findings do not imply malignant transformation. Rather, they reflect the involvement of central hub genes such as AKT1, EGFR, RELA (NF-κB p65), and MMP9 that regulate inflammation, cell proliferation, survival, and tissue remodeling, core biological processes shared by chronic inflammatory disorders and cancer-associated signaling pathways. Consistent with this interpretation, GO and pathway enrichment analysis revealed that the predicted target genes are predominantly associated with inflammatory regulation, cellular stress responses, and tissue repair mechanisms relevant to the pathophysiology of peptic ulcer disease.

Collectively, these computational findings provide plausible mechanistic hypotheses for the gastroprotective activity of EECE by describing the potential involvement of inflammatory signaling, epithelial stress responses, apoptosis, and mucosal repair pathways. Among the predicted hub genes, RELA (NF-κB p65) emerged as a prominent candidate that may integrate inflammatory signaling, cellular stress responses, and tissue remodeling within the predicted interaction network. However, these predicted compound–target interactions and signaling pathways are derived from network pharmacology analysis and should not be interpreted as direct evidence of causal molecular mechanisms. Therefore, the proposed pathways require further experimental validation through targeted molecular studies. Guided by these computational predictions, subsequent in vivo experiments were performed to evaluate the gastroprotective activity of EECE and to assess the modulation of NF-κB p65 expression as an initial validation of one of the predicted molecular targets. Accordingly, the subsequent in vivo study was designed to confirm the gastroprotective activity and to examine NF-κB p65 expression as an initial biological validation of one of the key targets predicted by network pharmacology.

The in vivo findings were consistent with the predicted biological processes associated with gastric mucosal protection. In the negative control group, ethanol administration induced extensive hemorrhagic lesions, elevated ulcer indices, and severe mucosal erosion, confirming successful establishment of gastric injury and establishing a relevant biological context for assessing the gastroprotective effects of EECE. Consistent with the known pathophysiology of ethanol-induced gastric damage, characterized by epithelial disruption, excessive oxidative stress, and inflammatory cell infiltration leading to hemorrhagic lesions and mucosal erosion [36].

Ulcer diameter, which reflects the localized severity and depth of mucosal damage, decreased after EECE administration, with greater reductions at higher doses, suggesting a dose-related protective effect on gastric mucosal integrity. The absence of a statistically significant reduction at 200 mg/kg BW suggests that this dose was insufficient to effectively limit lesion expansion. In contrast, doses of 400 and 800 mg/kg BW markedly suppressed ulcer progression. This response pattern highlights the importance of sufficient exposure to bioactive compounds in counteracting ethanol-induced mucosal disruption. Comparable gastroprotective effects, with greater protection observed at higher doses or concentrations, have also been reported for plant-derived extracts rich in polyphenols and other bioactive metabolites [72].

Interestingly, although sucralfate provided partial protection, it did not significantly reduce ulcer area or index ulcer compared with the negative control. This observation may be explained by the primarily cytoprotective mechanism of sucralfate, which forms a physical barrier over the ulcer surface, thereby protecting the mucosa from acid, pepsin, and bile salts without substantially modulating oxidative stress or inflammatory signaling pathways [73]. In contrast, EECE and quercetin significantly reduced ulcer area and severity, suggesting broader protective mechanisms beyond surface shielding.

The negative control group exhibited pronounced gastric hemorrhage and extensive mucosal lesions, reflecting severe ethanol-induced gastric injury. Treatment with sucralfate provided partial protection, as gastric lesions and hemorrhagic areas remained evident, consistent with its primary cytoprotective mechanism. In contrast, quercetin administration effectively prevented the formation of visible gastric lesions, although mild hemorrhagic areas remained. Notably, EECE treatment provided the most pronounced gastroprotective effect, with a complete absence of gastric lesions across all treatment doses; at 800 mg/kg BW, the gastric mucosa appeared intact and free of hemorrhage. These macroscopic observations are consistent with reductions in ulcer area, ulcer diameter, and ulcer index, as well as increased gastric protection percentages observed in EECE-treated groups following ethanol-induced injury.

The superior gastroprotective effects observed in the quercetin and EECE-treated groups can be attributed to their antioxidant and anti-inflammatory properties. Quercetin, a major flavonol identified in EECE, effectively scavenges reactive oxygen species and enhances endogenous antioxidant defenses. Oxidative stress plays a pivotal role in ethanol-induced gastric injury by promoting lipid peroxidation, epithelial apoptosis, and microvascular dysfunction. Therefore, the high protective indices observed in EECE-treated animals are likely mediated, at least in part, by suppression of oxidative damage and preservation of mucosal redox balance [15,74].

Moreover, increasing evidence suggests that modulation of inflammatory signaling pathways is crucial for effective gastroprotection. Ethanol exposure activates the NF-κB pathway, leading to the transcription of pro-inflammatory mediators such as TNF-α, IL-1β, and COX-2, thereby exacerbating mucosal injury and delaying healing [35]. The significant reduction in ulcer severity and the high protection percentages observed with EECE administration are consistent with inhibition of NF-κB-mediated inflammatory responses, as further supported by the observed downregulation of NF-κB p65 expression. This mechanistic link strengthens the biological relevance of EECE as an anti-ulcer agent.

Beyond flavonoids, UHPLC–HRMS/MS-based metabolomic profiling yielded putative annotations of several amino sugar-related metabolites in EECE, including N-Acetylquinovosamine and N-acetylglucosaminitol. These compounds are structurally related to N-acetylglucosamine, a key building block in the synthesis of mucin glycoproteins. Gastric mucus constitutes the first line of defense against luminal aggressors, and enhanced mucin production has been shown to improve mucosal resistance to ethanol-induced injury [51,52]. The presence of acetylated amino sugars in EECE may therefore contribute to mucin biosynthesis and reinforcement of the gastric mucus barrier, although these proposed effects were not directly evaluated in the present study.

In addition, EECE contained amino acid conjugates and small peptides, such as prolyl leucine, which are implicated in epithelial regeneration and tissue repair. Amino acids play essential roles in regulating epithelial proliferation, differentiation, and barrier integrity in the gastrointestinal tract, thereby facilitating mucosal healing following injury [75,76]. The reduced lesion areas observed macroscopically in EECE-treated groups may partially reflect not only protective effects but also accelerated mucosal recovery.

Lipid-derived metabolites, including oleamide, were also detected in EECE and may contribute to its gastroprotective activity. Based on previous studies, oleamide and related lipid amides have been reported to exert anti-inflammatory effects by suppressing iNOS and COX-2 expression and modulating NF-κB signaling [48,77]. Lipids further contribute to maintaining the hydrophobic properties of the gastric mucus layer, which are critical for preventing hydrogen-ion back-diffusion and preserving epithelial integrity [48]. These mechanisms collectively support the observed reduction in mucosal erosion and hemorrhage.

Although present at lower relative abundances, phenolic and indole derivatives, such as 3-Indoleacrylic acid, may further contribute to the antioxidant capacity of EECE. Ethanol-induced gastric injury is strongly associated with excessive ROS generation, leading to lipid peroxidation and inflammatory amplification. Even minor amounts of antioxidant metabolites may collectively help reduce oxidative stress [62], although their individual contributions and potential interactions were not investigated in the present study. Therefore, it is plausible that the combined presence of multiple metabolite classes contributes to the observed gastroprotective activity of EECE; however, this proposed interaction should be regarded as a hypothesis requiring further experimental validation.

Taken together, the phytochemical characterization and biological findings suggest that multiple classes of bioactive metabolites may collectively contribute to the gastroprotective activity of EECE rather than a single compound acting alone. Based on their reported biological activities, amino sugars may contribute to reinforcement of the gastric mucus barrier, lipid-derived metabolites and amino acid derivatives may support epithelial integrity and tissue repair, whereas flavonoids and phenolic compounds may help attenuate oxidative stress and inflammatory responses. However, the present study did not investigate the individual contributions, synergistic interactions, or specific molecular mechanisms of these metabolites. Future studies involving bioactivity-guided fractionation, isolation of individual compounds, and pathway-specific molecular investigations will be necessary to verify the contribution of each metabolite class to the gastroprotective effects of EECE.

To further evaluate the biological relevance of these proposed mechanisms at the tissue level, histopathological evaluation was performed to assess structural alterations and inflammatory cell infiltration in the gastric mucosa. The severe histopathological alterations observed in the negative control group confirm successful induction of ethanol-induced gastric injury. Morphologically, this damage was characterized by epithelial erosion, submucosal edema, and infiltration by acute inflammatory cells, particularly neutrophils (PMNs), reflecting disruption of the mucosal barrier and activation of the acute inflammatory response [78,79]. High PMN infiltration indicates the active involvement of inflammatory cells in the release of pro-inflammatory mediators and reactive oxygen species (ROS), which play critical roles in exacerbating tissue damage, increasing oxidative stress, and accelerating the degradation of the gastric mucosal protective layer [80,81].

The minimal histological alterations observed in the normal group indicate intact mucosal architecture and absence of inflammatory activation. The improvement observed in the sucralfate-treated group is consistent with its established cytoprotective mechanism, which involves the formation of a viscous protective layer over the gastric mucosa via polymerization reactions under acidic conditions [73]. This layer functions as a barrier against aggressive factors such as gastric acid, pepsin, and bile salts, while simultaneously creating a microenvironment that supports mucosal healing and tissue regeneration [82]. With reduced mucosal exposure to irritants, activation of local inflammatory pathways can be suppressed, as indicated by decreased pro-inflammatory mediators and improved epithelial barrier integrity [52]. This condition reduces chemotactic signals that attract neutrophils to the injury site, resulting in fewer PMNs migrating to and accumulating in the damaged gastric tissue.

Quercetin-mediated gastroprotection is likely attributable to its combined antioxidant and anti-inflammatory properties, as reflected by the marked reduction in PMN infiltration and the preservation of gastric mucosal thickness. These findings indicate that quercetin effectively limits neutrophil recruitment and attenuates oxidative stress in gastric tissue, thereby maintaining mucosal structural integrity and facilitating accelerated healing [83,84]. This effect can also be explained by the antioxidant activity of quercetin, which acts as a ROS scavenger, neutralizing free radicals and reducing oxidative stress, both of which play a crucial role in gastric mucosal injury [57]. The reduction in oxidative stress contributes to the preservation of epithelial integrity, as oxidative stress and lipid peroxidation are known to increase mucosal permeability and exacerbate tissue damage under ulcerative conditions [83]. In addition, quercetin exhibits anti-inflammatory properties by inhibiting the activation of inflammatory pathways, including suppression of NF-κB p65 activation and reduced expression of pro-inflammatory mediators, thereby attenuating excessive inflammatory responses in gastric tissue [85]. These mechanisms ultimately contribute to reduced accumulation of inflammatory cells, as evidenced by reduced infiltration in ethanol-induced gastric ulcer models [83].

EECE treatment demonstrated dose-dependent gastroprotective effects, with higher doses providing more pronounced histological improvement. Partial protection at lower doses suggests early suppression of inflammatory infiltration, whereas optimal protection at 800 mg/kg BW was associated with both reduced PMN accumulation and restoration of mucosal thickness. This observation aligns with previous reports indicating that attenuation of inflammatory responses often precedes structural regeneration of gastric tissue [35]. The reduction in PMN infiltration, accompanied by increased mucosal thickness, underscores the close relationship between inflammatory control and tissue healing. Previous studies have shown that suppression of neutrophil recruitment is associated with inhibition of inflammatory pathways, including NF-κB p65, which plays a crucial role in the pathogenesis of gastritis and gastric ulcers [86,87].

Overall, the integration of histopathological scoring, mucosal thickness, and PMN cell counts provides a comprehensive assessment of the extent of gastric mucosal injury and the gastroprotective effects of EECE. The significant reduction in PMN cell infiltration observed in the treated groups, particularly at higher EECE doses, suggests that attenuation of inflammatory responses may contribute to the reduction in tissue damage and the preservation of gastric mucosal architecture. These histopathological findings are biologically consistent with the inflammation-related biological processes predicted by the network pharmacology analysis, although they do not by themselves establish the underlying molecular mechanisms. NF-κB signaling is a well-established regulator of inflammatory cytokine production, neutrophil recruitment, and gastric mucosal injury during ethanol-induced ulceration [88,89]. Therefore, to further investigate one of the predicted molecular mechanisms underlying these histopathological improvements, NF-κB p65 expression was subsequently evaluated because of its established role in regulating inflammatory responses during gastric mucosal injury.

The increased expression of NF-κB p65 observed in the negative control group is consistent with the established mechanism of ethanol-induced gastric mucosal injury, which involves excessive ROS production and oxidative stress-mediated activation of NF-κB signaling. Activation of NF-κB p65 promotes the transcription of pro-inflammatory cytokines, including TNF-α, IL-1β, and IL-6, as well as inflammatory enzymes such as iNOS and COX-2, leading to inflammatory cell infiltration and exacerbation of gastric tissue damage [87,90]. Activation of NF-κB p65 has been widely recognized as a central event in the pathogenesis of ethanol-induced gastric mucosal injury, given its pivotal role in coordinating inflammatory responses and amplifying inflammatory signaling, culminating in progressive mucosal damage [39,88].

The reduced NF-κB p65 expression observed in the sucralfate-treated group suggests that, beyond its well-known cytoprotective action through formation of a physical barrier on the gastric mucosa, sucralfate may also exert anti-inflammatory effects by modulating NF-κB pathway activation. Inhibition of NF-κB signaling has been associated with decreased transcription of pro-inflammatory mediators that aggravate gastric mucosal injury [63,91,92]. Consistent with this, quercetin has been reported to attenuate gastric inflammatory responses by regulating the HMGB1/TLR4/NF-κB signaling pathway, thereby limiting amplification of inflammatory signals and the progression of mucosal injury in models of gastritis and gastric ulceration [93,94,95].

The dose-related reduction in NF-κB p65 expression following EECE administration suggests a modulatory effect on inflammatory signaling, particularly at higher doses. This observation aligns with previous studies reporting that natural compounds with anti-inflammatory properties can attenuate NF-κB p65 activation in experimental models of gastric ulceration [84,89]. Although changes in total NF-κB p65 protein expression did not reach statistical significance, the consistent downward trend is biologically relevant and may reflect increasing efficacy with higher exposure levels. In the pathophysiology of gastritis and gastric ulceration, modulation of NF-κB signaling is often more sensitively reflected in changes in pathway activation and downstream signaling events rather than in marked alterations in total protein expression, yet it still contributes to the attenuation of inflammatory responses [39,88].

Importantly, the observed trend of reduced NF-κB p65 expression is consistent with histopathological improvements, including decreased PMN infiltration, lower tissue injury scores, and restored mucosal thickness in the treatment groups. This concordance underscores the role of NF-κB p65 modulation in limiting inflammatory cell recruitment and restraining the progression of gastric mucosal injury. Collectively, these Western blot findings support the hypothesis that the gastroprotective effects of the treatments—particularly EECE—are mediated, at least in part, through suppression of inflammatory responses via regulation of the NF-κB p65 signaling pathway, even though these effects are not fully reflected as statistically significant changes in total protein expression [39,89]. Furthermore, although the network pharmacology analysis identified several potential therapeutic targets, only NF-κB p65 was experimentally validated in this study. Future investigations should validate additional predicted targets, including AKT1, PTGS2, and CASP3, to provide a more comprehensive understanding of the molecular mechanisms underlying the gastroprotective effects of EECE.

The present findings should also be interpreted in light of the source of the plant material. The *C. esculenta* corms used in this study were collected from a single geographical location during a single harvesting period. Since the phytochemical composition of medicinal plants may vary according to genotype, environmental conditions, cultivation practices, post-harvest handling, and harvest season, the chemical profile and gastroprotective activity observed in the present study may not fully represent the natural variability of the species. Future studies involving multiple geographical locations, harvesting periods, and independent plant batches are warranted to further evaluate the reproducibility and generalizability of these findings.

A limitation of this study is the reduced sample size in the final analysis due to the exclusion of extreme outliers likely resulting from technical variability during sample processing, which may have limited the statistical power. In addition, the ethanol-induced gastric ulcer model primarily reflects acute oxidative and inflammatory injury and does not fully recapitulate the pathophysiological mechanisms of *H. pylori*- or NSAID-induced ulcers. Furthermore, authentic standards and quantitative spectral matching scores were not available for the annotated metabolites; therefore, these compounds should be considered putative annotations rather than confirmed identifications. Although the present findings provide evidence for the gastroprotective effects of EECE, further studies using larger sample sizes, additional ulcer models, and authentic standards or complementary analytical approaches are warranted to confirm and extend these findings.

## 4. Materials and Methods

### 4.1. Plant Material Collection and Identification

Corms were collected from a taro plantation in Cikeas Village, Sukaraja Subdistrict, Bogor Regency, West Java, Indonesia (Google Maps coordinates: 6°31′21″ S, 106°51′01″ E). The plant samples were identified by Arifin Surya Dwipa Irsyam (Scopus ID: 57211286941) at the Herbarium Bandungense, School of Life Sciences and Technology, Bandung Institute of Technology, Indonesia, and were confirmed as *Colocasia esculenta* (L.). Scott (family Araceae) [96,97], with document number 2455/IT1.C11.2/TA.00/2025, signed by Angga Dwiartama, Ph.D. The plant specimen was not deposited in the Herbarium Bandungense.

### 4.2. Preparation of Corm Powder

Fresh corms were peeled, washed thoroughly under running water, and sliced to facilitate drying. The slices were sun-dried for 2 days (8 h per day), ground to a coarse powder, and sieved through a 60-mesh sieve (featuring 60 holes/linear inch). The resulting powder was stored in a tightly closed plastic container until extraction.

### 4.3. Preparation of Ethanol Extract (EECE)

Extraction was performed by briefly immersing the corm powder in 70% ethanol, prepared by diluting absolute ethanol (EMSURE^®^, Merck, Darmstadt, Germany; Cat. No. 1.00983), at a material-to-solvent ratio of 1:10 (*w*/*v*) for 3 × 24 h at room temperature, with occasional stirring during the first 6 h. The extracts were collected, filtered, and the solvent was removed by rotary evaporation at 40 °C, yielding a viscous extract (EECE). A single batch of EECE was prepared and used for all subsequent phytochemical analyses (TPC, TFC, HPLC, and UHPLC–HRMS/MS), network pharmacology, in vivo experiments, histopathological evaluation, and Western blot analysis to ensure consistency throughout the study.

### 4.4. Proximate Analysis

The proximate composition of EECE (moisture, lipid, ash, protein, carbohydrate) and the vitamin C content were determined. Moisture, lipid, ash, and protein were determined in accordance with SNI 01-2891:1992.

### 4.5. Total Phenol Content (TPC)

TPC was determined using the Folin–Ciocalteu reagent (Merck, Darmstadt, Germany; Cat. No. 1.09001), which quantifies phenolic compounds by reducing them to form a blue complex [98]. TPC was calculated using a gallic acid standard curve [99,100]. Briefly, EECE (0.2 g) was dissolved in methanol (EMSURE^®^, analytical grade, Merck, Darmstadt, Germany; Cat. No. 1.06007) and adjusted to a final volume of 25 mL. One mL of the sample or standard solution was mixed with 5.0 mL of diluted Folin–Ciocalteu reagent, then 4.0 mL of sodium carbonate solution (7.5%, *w*/*v*), prepared by dissolving sodium carbonate (Merck, Darmstadt, Germany) in distilled water, was added to the reaction mixture. After incubation, the absorbance was measured at 730 nm.

### 4.6. Total Flavonoid Content (TFC)

TFC was determined using the aluminum chloride (AlCl_3_) colorimetric method, as previously described. EECE (0.2 g) was dissolved in ethanol (EMSURE^®^, Merck, Darmstadt, Germany; Cat. No. 1.00983) and adjusted to a final volume of 25 mL. An aliquot of 0.5 mL of the sample or standard solution was mixed with 1.5 mL of ethanol, 0.1 mL of AlCl_3_ solution (10%, *w*/*v*) was prepared from aluminium chloride (AlCl_3_, Sigma-Aldrich, St. Louis, MO, USA; Cat. No. 206911), 0.1 mL of sodium acetate solution (1 M), which was prepared from sodium acetate (EMSURE^®^, Merck, Darmstadt, Germany; Cat. No. 1.06268), and 2.8 mL of distilled water. The reaction mixture was incubated at room temperature for 30 min, and the absorbance was measured at 510 nm using an ultraviolet-visible spectrophotometer. A blank solution without AlCl_3_ was prepared. TFC was calculated from the quercetin standard curve [101].

### 4.7. Determination of Quercetin in EECE Using RP-HPLC

Quercetin in EECE was identified and quantified by reverse-phase high-performance liquid chromatography (RP-HPLC) following recently validated methods [102,103]. Before injection, the sample and standard solutions were filtered through a membrane filter. Chromatographic separation was performed on an octadecylsilane C18 column (250 mm × 4.6 mm internal diameter) with a mobile phase of 0.1% trifluoroacetic acid (Sigma-Aldrich, St. Louis, MO, USA; Cat. No. T6508) in water [A], methanol (LiChrosolv^®^, gradient grade for HPLC, Merck, Darmstadt, Germany; Cat. No. 1.06035) [B], and acetonitrile (Fisher Scientific, Fair Lawn, NJ, USA; Cat. No. A996-4) [C], delivered at a flow rate of 1.0 mL/min. The injection volume was 10–20 μL, and detection was performed at the characteristic UV wavelength of quercetin (370 nm). Quantification was performed using a quercetin standard curve (Sigma-Aldrich, St. Louis, MO, USA; Cat. No. Q4951).

### 4.8. UHPLC-HRMS/MS-Based Metabolite Profiling

The chemical profile of EECE was analyzed using an ultra-high-performance liquid chromatography–high-resolution mass spectrometry tandem mass spectrometry (UHPLC–HRMS/MS) system with electrospray ionization in positive mode (ESI+). Chromatographic separation was performed using a Thermo Scientific Vanquish™ UHPLC Binary Pump (Thermo Fisher Scientific, Waltham, MA, USA) equipped with an Accucore™ Phenyl-Hexyl column ((100 mm × 2.1 mm, 2.6 µm; Thermo Fisher Scientific, Waltham, MA, USA) maintained at 40 °C. The mobile phase consisted of MS-grade water containing 0.1% (*v*/*v*) formic acid (A) and MS-grade methanol containing 0.1% (*v*/*v*) formic acid (B), delivered at a flow rate of 0.3 mL/min using gradient elution. Methanol (Merck, Darmstadt, Germany; Cat. No. 632546) and formic acid (Merck, Darmstadt, Germany; Cat. No. 1.59013) were used. The gradient program started at 5% B, increased linearly to 90% B over 16 min, held at 90% B for 4 min, and then returned to the initial condition (5% B) for 25 min to re-equilibrate the column.

The sample was prepared by dissolving 1 mg of EECE in 1 mL of 100% methanol (Merck, Darmstadt, Germany; Cat. No. 632546), and 3 µL was injected into the system. Mass spectrometric detection was carried out using a Q Exactive™ Hybrid Quadrupole-Orbitrap™ high-resolution mass spectrometer (Thermo Fisher Scientific, Waltham, MA, USA) operated in ESI+ mode. The capillary voltage was set to 3.30 kV, the capillary temperature to 320 °C, and mass spectra were acquired over an *m*/*z* range of 66.7–1000. Metabolite annotation was performed by comparing chromatographic retention time, accurate precursor mass, and MS/MS fragmentation spectra with the mzCloud Mass Spectral Library. Candidate metabolites were further cross-checked against the ChemSpider and PubChem databases. The reported metabolites represent putative annotations rather than confirmed identifications, as authentic reference standards, retention-time confirmation with reference compounds, and quantitative spectral matching scores were not available.

### 4.9. Network Pharmacology Analysis

Active compounds identified by UHPLC-HRMS/MS were subjected to target prediction using open-access databases. Disease-related targets for PUD were retrieved from the GeneCards database. Common targets shared by compounds and disease were identified using intersection analysis. Protein–protein interaction (PPI) networks were constructed using the STRING database (confidence score ≥ 0.4) and visualized in Cytoscape (version 3.10.4). Core targets were identified based on topological parameters, including degree and betweenness centrality. Gene Ontology (GO) and Kyoto Encyclopedia of Genes and Genomes (KEGG) pathway enrichment analyses were performed using Metascape (version 3.5.20260701), with significant terms defined by adjusted *p*-values < 0.05.

### 4.10. In Vivo Gastroprotective Study

#### 4.10.1. Animal Ethics, Handling, and Maintenance

The study was conducted on male Wistar rats. The protocol for animal handling was approved on 8 August 2025 by the Research Ethics Committee of Universitas Padjadjaran, Indonesia (approval document number 687/UN6.KEP/EC/2025, signed by Dr. Muhammad Hasan Bashari). The procedure was carried out by strictly adhering to The Guide for the Care and Use of Laboratory Animals (NRC 2011; eighth edition) (https://grants.nih.gov/grants/olaw/guide-for-the-care-and-use-of-laboratory-animals.pdf accessed on 21 May 2025) (Guide for the Care and Use of Laboratory Animals 2011), and The ARRIVE guidelines 2.0 Animal Research: Reporting of In Vivo Experiments (https://arriveguidelines.org/arrive-guidelines accessed on 21 May 2025). The procedures were carried out at the Pharmacology Laboratory, Faculty of Pharmacy, Universitas Padjadjaran, Indonesia.

Male Wistar rats with an approximate body weight of 180 g were purchased from the Animal Breeding Facility, Division of Animal Laboratories of PT. Biofarma, Jl. Kolonel Masturi Kav 10 Kertawangi, Cisarua, West Java, Indonesia, and housed under controlled environmental conditions (22–25 °C; 50–60% relative humidity), with a 12 h light/12 h dark cycle. Animals were kept at a density of 5 rats per cage, fed a standard rodent chow (containing approximately 18% crude protein and 5% fat), and given free access to drinking water. All animals were acclimatized for 7 days before the experiment.

#### 4.10.2. Experimental Grouping and Treatments

Rats were randomly allocated into seven groups. Group 1: normal control-0.5% Na-CMC for 14 days; Group 2: negative control-ethanol induced PUD and 0.5% Na-CMC for 14 days; Group 3: ethanol-induced PUD and sucralfate 270 mg/kg in 0.5% Na-CMC for 14 days; Group 4: ethanol-induced PUD and quercetin 15 mg/kg in 0.5% Na-CMC for 14 days; Group 5: ethanol-induced PUD and EECE 200 mg/kg in 0.5% Na-CMC for 14 days; Group 6: ethanol-induced PUD and EECE 400 mg/kg in 0.5% Na-CMC for 14 days; Group 7: ethanol-induced PUD and EECE 800 mg/kg in 0.5% Na-CMC for 14 days.

Treatment with sucralfate (Meprofarm, Bandung, Indonesia), quercetin (Sigma-Aldrich, St. Louis, MO, USA; Cat. No. Q4951), or EECE was administered via oral gavage for 14 days. On day 14 (D14), all rats received their respective treatments. One hour after treatment administration, PUD was induced by oral administration of 70% ethanol, prepared by diluting absolute ethanol (EMSURE^®^, Merck, Darmstadt, Germany; Cat. No. 1.00983) (5 mL/kg body weight) to all groups except the normal control. One hour after ethanol administration, rats were euthanized under anesthesia according to institutional procedures, using a combination of ketamine (125 mg/kg body weight) and xylazine (10 mg/kg body weight) administered intraperitoneally. When rats were fully anesthetized, euthanasia was performed by trained personnel through cervical dislocation, and the stomachs were collected for evaluation. The remains were wrapped in medical waste plastics and buried in an animal waste burial.

#### 4.10.3. Macroscopic Evaluation and Ulcer Index

The stomachs were excised, opened along the greater curvature, gently rinsed with 0.9% NaCl solution (Otsuka, Jakarta, Indonesia), spread flat, and the gastric mucosa was examined for hemorrhagic lesions [104]. Lesion severity was scored using a standardized scale [35]. The ulcer index (UI) was calculated as the total ulcer score divided by the number of animals presenting gastric lesions. The percentage of protection (protection ratio) was calculated using the ulcer index values of the control and treated groups, following a previous protocol by Oloyede et al. [105].

$$Protection\left(\%\right)=\frac{UIcontrol-UItreated}{UIcontrol}\times 100$$

#### 4.10.4. Histopathological Examination

Gastric tissues were fixed in 10% neutral buffered formalin (Indopath, Jakarta, Indonesia; Cat. No. IPPN05) for 24 h, dehydrated in graded ethanol (Merck, Darmstadt, Germany; Cat. No. 1.00983), cleared in xylene (Merck, Darmstadt, Germany; Cat. No. 1.08633), infiltrated with parrafin (Merck, Darmstadt, Germany; Cat. No. 1.07158), embedded, and sectioned (3–5 μm). Sections were stained with hematoxylin and eosin (H&E) (Merck, Darmstadt, Germany; Cat. Nos. 1.05174 and 1.15935) and examined under light microscopy (100× for general morphology; 400× for inflammatory cell assessment). Histological injury (epithelial loss, edema, hemorrhage, inflammatory infiltration, and lamina propria erosion) was scored according to established criteria [35,106]. The number of polymorphonuclear neutrophils (PMNs) was quantified by counting cells in five randomly selected high-power fields (HPFs) at 400× magnification and expressed as cells per HPF (cells/HPF).

#### 4.10.5. Western Blot Analysis of NF-κB p65 Expression

Fresh gastric tissue (~25 mg) was homogenized in SDS lysis buffer containing Tris Base (Servicebio, Wuhan, China), Glycine (Servicebio, Wuhan, China), and 0.1% Tween-20 (Sigma-Aldrich, Darmstadt, Germany), and centrifuged at 15,000 rpm for 5 min. Protein samples were prepared with sample buffer (1:1), heated at 95 °C for 5 min, separated by SDS-PAGE using a 15% separating gel and stacking gel with TEMED (Sigma-Aldrich, Darmstadt, Germany), and transferred onto a nitrocellulose membrane at 200 mA for 30 min. Membranes were stained with Ponceau S solution (Sigma-Aldrich, Darmstadt, Germany), washed with PBST, and blocked with 5% BSA (Sigma-Aldrich, Darmstadt, Germany). Membranes were incubated overnight at 4 °C with primary antibodies against NF-κB p65 (ABclonal Technology, Wuhan, China; Cat. No. A19653, 1:1000) and β-actin (Minneapolis, MN, USA; Cat. No. MAB8529, 1:1000), the membranes were incubated with goat anti-mouse IRDye^®^ 680RD and goat anti-rabbit IRDye^®^ 800CW secondary antibodies (LI-COR Biosciences, Lincoln, NE, USA; Cat. Nos. 925-32210 and 926-32211) at a dilution of 1:15,000 for 2 h at room temperature. The molecular weight marker used was the BioHelix Prestained Protein Ladder PMB11-500 (BioHelix, Cat. No. PMB11-500).

The protein loading control was β-actin, with 20 µg of total protein loaded per lane. Western blot analysis was performed using two replicates per group. Protein bands were visualized using the LI-COR Odyssey CLx Imaging System (LI-COR Biosciences) with a 6 min exposure time on the 800 nm channel. Band intensities were quantified using Image Studio software (version 5.2; LI-COR Biosciences) [107], and NF-κB p65 band intensities were normalized to the corresponding β-actin band intensities.

### 4.11. Statistical Analysis

Data were analyzed using SPSS v30. Normality was assessed before hypothesis testing. Normally distributed data were analyzed using one-way ANOVA followed by the LSD post hoc test. Non-normally distributed data were analyzed using Kruskal–Wallis followed by the Mann–Whitney test. Differences were considered statistically significant at *p* < 0.05.

## 5. Conclusions

EECE demonstrated marked gastroprotective activity against ethanol-induced gastric injury by effectively preserving gastric mucosal integrity and attenuating inflammatory damage. Macroscopic and histopathological evaluations confirmed significant reductions in ulcer severity and PMN cell infiltration following EECE treatment. Mechanistically, EECE suppressed NF-κB p65 activation, consistent with network pharmacology analysis, which identified RELA, the gene encoding NF-κB p65, as a key regulatory target associated with PUD. In addition, UHPLC-HRMS/MS profiling revealed bioactive metabolites with anti-inflammatory and antioxidant properties that may synergistically contribute to mucosal protection. Overall, these findings establish EECE as a promising natural gastroprotective agent against inflammation-mediated gastric mucosal injury.

## Figures and Tables

**Table 1:** Proximate composition of *C. esculenta* crude dried corm powder and EECE.

Proximate Composition	Content (%)	
	*C. esculenta* Corm Powder	Ethanol Extract of *C. esculenta* Corm
Moisture	9.61	28.93
Ash	2.94	44.22
Lipid	0.51	8.56
Protein	5.82	7.27
Carbohydrate	81.12	11.02

**Table 2:** Putatively annotated major metabolites in the ethanolic extract of *Colocasia esculenta* (EECE) detected by UPLC-HRMS/MS analysis.

Metabolite Annotation	Molecular Formula	Exact Mass	Retention Time (RT)	MS/MS Fragments (*m*/*z*)	Relative Abundance (%)
N-(1-deoxy-1-fructosyl)phenylalanine	C_15_H_21_NO_7_	327.13185	1.432	91.0546; 97.0287; 120.0810; 132.0810; 166.0865; 216.1014; 264.1233; 292.1182; 310.1285	10.341
1-[(3-carboxypropyl)amino]-1-deoxyfructose	C_10_H_19_NO_7_	265.11647	0.868	87.0444; 98.0604; 104.1073; 170.0817; 182.0813; 230.1024; 248.1130	9.695
N-(1-deoxy-1-fructosyl)leucine	C_12_H_23_NO_7_	293.14729	1.108	86.0968; 88.0397; 97.0287; 132.1020; 212.1280; 230.1386; 258.1338; 276.1444	6.404
N-Acetylquinovosamine	C_8_H_15_NO_5_	205.09505	0.842	69.0338; 86.0605; 97.0287; 126.0552; 158.0814; 188.0917	5.771
α-PALMITIN	C_19_H_38_O_4_	330.27656	14.45	57.0705; 71.0860; 81.0704; 95.0859; 123.1173; 221.2274; 239.2378; 313.2737	3.435
N-(1-carboxy-2-methylpropyl)glutamic acid	C_10_H_17_NO_6_	247.10590	0.791	87.0445; 97.0288; 98.0603; 116.0708; 182.0813; 230.1022	2.633
Adenosine	C_10_H_13_N_5_O_4_	267.09731	1.093	61.8298; 111.5800; 136.0620; 218.7926; 220.7688; 232.0824	2.223
N-(1-deoxy-1-fructosyl)valine	C_11_H_21_NO_7_	279.13207	0.911	72.0813; 84.0812; 118.0865; 130.0865; 216.1231; 244.1181; 262.1288	1.635
N-Acetylglucosaminitol	C_8_H_17_NO_6_	223.10556	0.85	74.0605; 86.0604; 97.0288; 128.0709; 158.0814; 188.0918; 206.1023	1.556
Oleamide	C_18_H_35_NO	281.27165	14.92	69.0704; 97.1015; 121.1013; 135.1168; 149.1327; 191.1798; 247.2423; 265.2530	1.474
3-Indoleacrylic acid	C_11_H_9_NO_2_	187.06360	2.405	53.9885; 91.0545; 115.0543; 117.0702; 118.0654; 144.0809; 146.0602	1.355
Adenine	C_5_H_5_N	135.05466	1.08	91.0545; 92.0247; 94.0403; 109.0510; 119.0354	1.335
Lotaustralin	C_11_H_19_NO_6_	261.12163	0.803	72.0813, 84.0812, 118.0865, 180.1017, 198.1124, 216.1229, 244.1182	0.857
Hexadecanamide	C_16_H_33_NO	255.25636	14.498	57.0704; 88.0760; 102.0917; 116.1070; 130.1239	0.408
Prolylleucine	C_11_H_20_N_2_O_3_	228.14752	1.098	70.0656; 72.0813; 142.0864; 211.1077	0.218

**Table 3:** Quantitative assessment of gastric ulcer indices and protection percentage following EECE.

Treatment Group	Relative Gastric Weight ± SD	Ulcer Area (%) ± SD	Ulcer Diameter (mm) ± SD	Ulcer Index ± SD	Protection (%)
Normal Control	0.75 ± 0.20	0.00 ± 0.00 *	0.00 ± 0.00 *	0.00 ± 0.00 *	100.00
Negative Control	0.86 ± 0.03	4.38 ± 1.20	2.17 ± 0.39	3.22 ± 0.79	-
Sucralfate	0.78 ± 0.07	1.41 ± 1.95	0.85 ± 1.21	1.11 ± 1.57	65.49
Quercetin	0.79 ± 0.06	0.14 ± 0.11 *	0.33 ± 0.24 *	0.56 ± 0.42 *	82.75
EECE 200 mg/kg	0.85 ± 0.16	1.28 ± 0.53 *	1.16 ± 0.87	0.67 ± 0.47 *	79.30
EECE 400 mg/kg	0.73 ± 0.04	0.09 ± 0.08 *	0.23 ± 0.32 *	0.33 ± 0.47 *	89.65
EECE 800 mg/kg	0.73 ± 0.08	0.01 ± 0.01 *	0.18 ± 0.26 *	0.11 ± 0.16 *	96.55

Abbreviations: EECE, ethanolic extract of *Colocasia esculenta*; SD, standard deviation. Data are presented as median (interquartile range), *n* = 3. Statistical analysis was performed using the Kruskal–Wallis test followed by the Mann–Whitney U test. An asterisk (*) indicates a statistically significant difference compared with the negative control group (*p* < 0.05).

**Table 4:** Histopathological parameters of gastric tissue in ethanol-induced rats.

Treatment Group	Ulcer Score ± SD	Gastric Mucosal Thickness (µm) ± SD	Number of PMN Cells ± SD
Normal Control	1.0 ± 0.8	1111.51 ± 136.73	2.4 ± 1.2 *
Negative Control	6.0 ± 1.6	697.23 ± 91.41	24.1 ± 0.5
Sucralfate	1.9 ± 0.7	1002.04 ± 116.44	0.7 ± 0.1 *
Quercetin	1.3 ± 0.7	1105.05 ± 41.84	7.1 ± 0.3 *
EECE 200 mg/kg	3.3 ± 1.3	929.07 ± 38.32	10.6 ± 2.2 *
EECE 400 mg/kg	2.5 ± 0.1	1054.01 ± 35.69	10.2 ± 1.6 *
EECE 800 mg/kg	1.8 ± 1.0	1061.71 ± 17.29	5.3 ± 0.9 *

Abbreviations: EECE, ethanolic extract of *Colocasia esculenta*; PMN, Polymorphonuclear; SD, standard deviation. Data are presented as median (interquartile range). Statistical analysis was performed using the Kruskal–Wallis test followed by the Mann–Whitney U test. An asterisk (*) indicates a statistically significant difference compared with the negative control group (*p* < 0.05).

## Data Availability

The datasets generated and analyzed during the current study are publicly available in the Science Data Bank. The raw polymorphonuclear (PMN) cell count data are available at https://doi.org/10.57760/sciencedb.27807, and the full, uncropped Western blot images are available at https://doi.org/10.57760/sciencedb.27808.

## Supplementary Materials

The following supporting information can be downloaded at: https://www.mdpi.com/article/10.3390/ijms27177693/s1.

## Abbreviations

The following abbreviations are used in this manuscript:

EECE	Ethanolic Extract of *Colocasia esculenta*
PUD	Peptic Ulcer Disease
NF-κB	Nuclear Factor Kappa B
TPC	Total Phenolic Content
TFC	Total Flavonoid Content
GAE	Gallic Acid Equivalent
QE	Quercetin Equivalent
UHPLC-HRMS	Ultra-High-Performance Liquid Chromatography–High-Resolution Mass Spectrometry
RT	Retention Time
LC-MS	Liquid Chromatography–Mass Spectrometry
NSAIDs	Nonsteroidal Anti-Inflammatory Drugs
Na-CMC	Sodium Carboxymethyl Cellulose
GSH	Reduced Glutathione
PMN	Polymorphonuclear Neutrophils
ROS	Reactive Oxygen Species
SOD	Superoxide Dismutase
CAT	Catalase
TLR4	Toll-Like Receptor 4
COX	Cyclooxygenase
PGE_2_	Prostaglandin E_2_
WB	Western Blot
IHC	Immunohistochemistry
H&E	Hematoxylin and Eosin
HPF	High-power fields
SDS-PAGE	Sodium Dodecyl Sulfate–Polyacrylamide Gel Electrophoresis
PBST	Phosphate-Buffered Saline with Tween 20
BSA	Bovine Serum Albumin
CASP3	Caspase-3
AKT1	AKT Serine/Threonine Kinase 1
EGFR	Epidermal Growth Factor Receptor
MMP9	Matrix Metallopeptidase 9
PTGS2	Prostaglandin-Endoperoxide Synthase 2
PPARG	Peroxisome Proliferator-Activated Receptor Gamma
RELA	RELA Proto-Oncogene, NF-κB Subunit (p65)
CASP8	Caspase-8
CASP9	Caspase-9
