# Supplementary material for: Colocasia esculenta Corm Extract Attenuates Ethanol-Induced Gastric Ulcer by Suppressing NF-κB Expression: An Integrated Network Pharmacology Approach and In Vivo Validation
Source: Int J Mol Sci. 2026 Aug 27;27(17):7693. doi: 10.3390/ijms27177693 (PMC13566257; doi:10.3390/ijms27177693)
Supplement: Supplementary file 1 [file ijms-27-07693-s001.zip › ijms-4464739-supplementary.pdf]

Table S1. Putative Metabolite Annotation Profile of EECE Obtained by UPLC–HRMS Analysis.

No	Metabolite annotation	Molecular formula	Exact mass	Retention time (RT)	Relative abundance (%)
1	N-(1-deoxy-1-fructosyl)phenylalanine	C15 H21 N O7	327.13185	1.432	10.341
2	1-[(3-carboxypropyl)amino]-1-deoxyfructose	C10 H19 N O7	265.11647	0.868	9.695
3	N-(1-deoxy-1-fructosyl)leucine	C12 H23 N O7	293.14729	1.108	6.404
4	N-Acetylquinovosamine	C8 H15 N O5	205.09505	0.842	5.771
5	α-PALMITIN	C19 H38 O4	330.27656	14.450	3.435
6	N-(1-carboxy-2-methylpropyl)glutamic acid	C10 H17 N O6	247.10590	0.791	2.633
7	Methyl isonicotinate	C7 H7 N O2	137.04773	0.895	2.499
8	N-(3-Carboxypropanoyl)-5-hydroxynorvaline	C9 H15 N O6	233.09001	0.889	2.498
9	1-Stearoylglycerol	C21 H42 O4	358.30829	15.516	2.378
10	Adenosine	C10 H13 N5 O4	267.09731	1.093	2.223
11	N-(1-deoxy-1-fructosyl)valine	C11 H21 N O7	279.13207	0.911	1.635
12	N-Acetylglucosaminitol	C8 H17 N O6	223.10556	0.850	1.556
13	Oleamide	C18 H35 N O	281.27165	14.920	1.474
14	5-aminosalicyluric acid	C9 H10 N2 O4	210.06403	0.896	1.422
15	3-Hydroxybutyrylcarnitine	C11 H21 N O5	247.14204	1.109	1.414
16	2-Deoxy-2,3-dehydro-N-acetylneuraminic acid	C11 H17 N O8	291.09569	0.905	1.364
17	3-Indoleacrylic acid	C11 H9 N O2	187.06360	2.405	1.355
18	Adenine	C5 H5 N	135.05466	1.080	1.335
19	Phenylalanine	C9 H11 N O2	165.07915	1.304	0.977
20	1-(ribofuranosyl)thymine	C10 H14 N2 O6	258.08550	0.770	0.915
21	Lotaustralin	C11 H19 N O6	261.12163	0,803	0.857
22	6-(glucosaminyl)-myo-inositol	C12 H23 N O10	341.13176	0.725	0.811
23	Glutamic acid	C5 H9 N O4	147.05313	0.888	0.788
24	1-Hydroxy-5-(4-methoxyphenyl)-3-pentanone	C12 H16 O3	208.11016	8.129	0.623
25	N-(1-deoxy-1-fructosyl)histidine	C12 H19 N3 O7	317.12212	0.862	0.539
26	Arginine	C6 H14 N4 O2	174.11173	0.724	0.448
27	Stearamide	C18 H37 N O	283.28746	15.929	0.421
28	Hexadecanamide	C16 H33 N O	255.25636	14.498	0.408
29	N,N-Diethyldodecanamide	C16 H33 N O	255.25636	15.841	0.295
30	Capryloylglycine	C10 H19 N O3	201.13658	1.104	0.249
31	Prolylleucine	C11 H20 N2 O3	228.14752	1.098	0.218
32	Muscone	C16 H30 O	238.22962	14.451	0.217
33	Libalyl propionate	C13 H22 O2	210.16210	6.455	0.208
34	Tyrosine	C9 H11 N O3	181.07408	1.093	0.192
